# Effects of resistance training on self-reported disability in older adults with functional limitations or disability – a systematic review and meta-analysis

**DOI:** 10.1186/s11556-019-0230-5

**Published:** 2019-12-07

**Authors:** Pia Øllgaard Olsen, Anne-Ditte Termannsen, Maja Bramming, Mark A. Tully, Paolo Caserotti

**Affiliations:** 1 Center for Active and Healthy Ageing, Department of Sports Science and Clinical Biomechanics, University of Southern Denmark Campusvej 55, DK-5230 Odense M, Denmark; 2 Nutrition and Health, University College Copenhagen, Sigurdsgade 26, 2200 Copenhagen N, Denmark; 3 National Institute of Public Health, University of Southern Denmark, Studiestræde 6, 1455 Copenhagen K, Denmark; 4 School of Health Sciences, Institute of Mental Health Sciences, Ulster University, Shore Road, Newtownabbey, Co Antrim BT37 0QB UK

**Keywords:** Disability, Aged, Resistance training, Activities of daily living, Needs assessment, Participation

## Abstract

**Background:**

Self-reported disability has a strong negative impact on older people’s quality of life and is often associated with the need for assistance and health care services. Resistance training (RT) has been repeatedly shown to improve muscle function (e.g. strength) and functional capacity (e.g. gait speed, chair-rise) in older adults with functional limitations. Nevertheless, it is unclear whether such objectively assessed improvements translate into a reduction in self-reported disability.

**Objectives:**

To assess: i) whether and to what extent RT interventions have an effect on self-reported disability in older adults (≥65 years) with functional limitations or disability; and ii) whether the effects on self-reported disability are associated with changes in objective measures of muscle strength and functional capacity across studies.

**Methods:**

PubMed, Embase, Web of Science, CINAHL and SPORTDiscus electronic databases were searched in June 2018. Randomized controlled trials reporting effects of RT on self-reported disability/function in ≥65 year-old adults with defined, functional limitations or self-reported disability were eligible. Data on self-reported disability/function were pooled by calculating adjusted standardized mean differences (SMD) using Hedges’g. Likewise, effect sizes for three secondary outcomes: knee extensor muscle strength; gait capacity; and lower body functional capacity were calculated and fit as covariates in separate meta-regressions with self-reported disability as the dependent factor.

**Results:**

Fourteen RCTs were eligible for the primary meta-analysis on self-reported disability. The total number of participants was 651 (intervention *n* = 354; control *n* = 297). A significant moderate positive effect of RT was found (SMD: 0.59, 95% CI: 0.253 to 0.925, *p* = 0.001). Between-study heterogeneity was present (I^2^ statistic = 75,1%, *p* <  0.001). RT effects on objective measures of lower body functional capacity were significantly associated with effects on self-reported disability (Adj. R^2^ = 99%, *p* = 0.002, *n* = 12 studies), whereas no significant associations with gait capacity or knee extensor strength were found.

**Conclusions:**

This review provides evidence that RT has a moderate positive effect on self-reported disability/function in old people with or at risk for disability. The effects are strongly associated with effects on objective measures of lower body functional capacity.

## Background

The prevalence of disability increases with increasing age [1] and is a serious societal challenge because of the estimated demographic trends towards an ageing population [2]. In European countries, the increased cost related to the additional need for assistance with activities of daily living (ADL) and long-term institutionalization of older people are projected to rise by 1.1% of gross domestic product between 2013 and 2060 [3].

Disability has been defined as a deficit between the capacities of an individual and that individual’s contextual factors [4]. Self-reported disability reflects an individual’s perception of this relationship, and refers to experienced difficulties in executing a task or being involved in a socially defined role including household, self-care and social life. Self-reported measures of disability and function in ADL reflects dependency of assistance, which is linked to reduced quality of life in older adults [5]. Moreover, older adult’s perception of function and disability play a role in the allocation of health care services. Health care providers commonly use interviews or questionnaires when rating older person’s need for assistance in basic ADL (eating, dressing, bathing, transferring from bed to chair and using the toilet) as well as more complex activities required for independent living such as housekeeping, cooking and shopping (instrumental ADL; IADL) [6]. Self-reported disability is therefore a highly relevant outcome which impacts the quality of life of the individual and challenges the sustainability of the health care sector [3, 7]. Preventing disability and maintaining independent living are therefore two high priorities in global health strategies [8].

Resistance training (RT), defined as exercise that causes muscles to work or hold against an applied force or weight [9], has been consistently reported to improve neuromuscular function (e.g. muscle strength) [10–13] and functional capacity (e.g. gait speed, chair-rise) [13–19] in older adults. These findings draw attention to RT as a means to preventing disability and dependency [10], and current health-recommendations encourage older adults to engage in RT on a weekly basis [9, 20]. Nevertheless, whether improvements in neuromuscular function and functional capacity translate into reduced self-reported disability in older adults is yet not conclusively established. This mismatch has been investigated in a randomized controlled trial (RCT) by Chandler et al. [16] in 1998. Their study showed a relationship between gains in strength and functional capacity (i.e. gait speed), but not between strength and self-reported disability after 10 weeks of RT [16]. A similar mismatch was later confirmed in older women with coronary heart disease by Brochu et al. [21]. The authors found a correlation between strength gain and functional capacity improvements, but not between strength gain and self-reported ADL-function [21]. While RT-interventions have often used assessments of neuromuscular function (e.g. muscle strength) and objective measures of functional capacity such as gait, chair-rise, reaching, stooping, lifting, [6, 14, 22], self-reported disability outcomes have received less attention and have shown inconsistent results [14, 23, 24]. In a systematic review, Weening-Dijksterhuis and colleagues [24] found that resistance-type exercise of moderate to high intensity had light to moderate effects on ADL-disability in frail institutionalized elderly (effects sizes < 0.50). In line with that, a Cochrane review and meta-analysis by Liu and Latham [23] showed that the effects of RT on self-reported disability measured by the functional domain of the 36-Item Short Form Health Survey Instrument (SF-36) and self-reported measures of ADL were significant, but small (33 trials, 2172 participants; SMD 0.14, 95% CI 0.05 to 0.22) [23]. However, the study populations in this comprehensive Cochrane review were heterogeneous in terms of health, functional status and age [23].

Ceiling effect has been suggested as a potential explanation for why RT-interventions fail to detect changes in self-reported ADL-disability in relatively well-functioning older adults [22]. Improvements in self-reported disability following RT-interventions may therefore only be detected in older adults with limitations or existing disability.

Understanding whether RT-interventions may translate into better self-perception of ability in participants with pre-existing limitations is therefore highly valuable for older adults, health care sector as well as for recommendation guidelines.

### Aims

The primary aim of this systematic review and meta-analysis is to investigate the effects of RT on self-reported disability in older people from the age of 65 years and above with functional limitations or self-reported disability. The secondary aim is to assess associations between the effects of RT on objective and subjective measures of disability across studies.

## Study design and methods

This literature review was conducted according to the PRISMA guidelines for systematic reviews and meta-analysis [25, 26]. The criteria of eligibility were specified in accordance to the PICOS approach (participants, intervention, comparison, outcomes, study design) as recommended by Centre for Evidence-Based Medicine [27] (details of the search strategy are outlined in Additional file 1). The quality assessment of eligible records was based on the validated tool for quality appraisal for reviews of physical therapy interventions – the Physiotherapy Evidence Database scale (PEDro scale) [28].

### Eligibility criteria

#### Participants/study population

Studies including participants from the age of 65 years and above and of any residential status, sex and ethnicity were eligible for inclusion. To ensure that all studies only included participants who met the age criterion, those that enrolled participants < 65 years were excluded, even if the sample mean age was > 65 years. The study population had to be characterized by having functional limitations (e.g. low gait speed) or self-reported disability according to a given criteria set by the authors of the original paper. Studies examining the impact of RT in populations with significant medical conditions such as cancer, renal and hepatic diseases, obstructive pulmonary disease, or neural abnormalities were not eligible. Additionally, study samples characterized by cognitive impairments, amputation or permanent use of wheelchair were excluded.

#### Intervention

To be considered for inclusion, RT needed to be the dominant component of the exercise intervention, with more than 50% of the intervention involving RT. The warm up and cool down periods were not considered as a part of the intervention and therefore the use of aerobic training, stretching, functional training and balance exercises during these phases was not used as the basis for exclusion. Interventions of any frequency, intensity and duration were eligible. Trials applying multifaceted interventions combining RT with macro-nutritional, behavioural, psychological, or medical approaches in all of the experimental groups were excluded as this could impact the results. Also, early rehabilitation interventions after joint surgery were ineligible since the influence of the natural post-surgery recovery process, has been identified as a confounding factor [29].

#### Comparison

Trials were eligible if they comprised at least one intervention group (IG) receiving an intervention that fulfilled the above listed criteria, and a control group (CG) that: i) did not receive any treatment or ii) was provided attention control, standard therapy, sham intervention or usual care. In case of the latter two, trials were eligible only if it was explicitly stated in the original article that this control treatment was expected to have no effect on the outcome measures.

#### Outcomes

This study adopted the language of the International Classification of Functioning, Disability and Health (ICF) [4] as recommended by international health policy makers and the research community [30]. The World Health Organisation (WHO) clearly states that the activity component (i.e. execution of a task or action) and the participation component (i.e. involvement in a life situation) in the ICF-model are overlapping [4]. The lack of clear cut definitions between the domains has been identified as a shortcoming of the ICF-model when used in research [30]. To enable a clear definition of outcomes in this review, the following categorization of outcomes was used.

##### Primary outcome

- Self-reported function/disability: measures aiming to quantify either the degree of functioning or disability in an individual in his/her life setting. These measures should be obtained from questionnaires, either self-administered or by interview, or by proxy (observation) as this approach is common in studies of the oldest old and institutionalized.

##### Secondary outcomes

- Functional capacity/limitation: objective measures of activities such as gait, stair climb, chair-rise and various imitated ADLs and IADLs, like transferring, personal care, and household obtained under standardized circumstances.
- Muscle function/impairment: measures of strength, power and muscle mass.

The primary outcome of the present review is self-reported disability. Trials were eligible if self-reported disability was measured by specific disability-questionnaires or by a subscale within questionnaires comprising multiple aspects of health status or health related quality of life. If the data from the relevant subscale were not presented separately in the original article, authors were contacted. In cases where more than one eligible measure of disability was presented in a trial, the measure with most items reflecting ADL and IADL was selected for analysis. Eligible outcomes could also include items related to mobility-disability.

#### Trial design

Only RCTs were eligible for inclusion. Trials comprising more than two study arms were eligible if the relevant outcome data were provided in the article separately for all groups, or if it could be obtained by request.

### Information sources and searches

In order to ensure an optimal search strategy for inclusion of all relevant records, a pilot search matrix was drafted and continually edited from December 2015 to October 2016 by two reviewers (PO and ADT). Subsequently a final comprehensive, generic matrix was developed and adapted for each database (Additional file 1). It comprised the following key search terms: older adults, resistance training, and self-reported ADL-disability representing participants, interventions and outcomes respectively, in accordance with the PICOS. Blocks of relevant synonyms for each key term were created, and subsequently combined by using Boolean operators. The electronic databases PubMed, Embase, Web of Science, CINAHL and SPORTdiscus were searched on June 27th, 2018. The title and abstract fields were searched in all databases. Moreover the “key words” field and relevant subject headings were searched when applicable. Articles of any western language and publication date were eligible.

### Study selection

Upon completion of the search, results were combined in EndNote X7.7.1 and duplicates were removed. The remaining records were then transferred to a web based software for review management (Covidence Veritas Health Innovation Ltd., 2019 [31]) and two reviewers (PO and MB) screened titles and abstracts to identify potentially eligible trials for full text assessment. PO and MB independently included or excluded records according to defined criteria. In the case of disagreements on any level in the selection process, PO and MB discussed the inclusion of a trial, until consensus was reached or a third reviewer (PC) was consulted.

### Data collection and extraction

PO, ADT and MB carried out the quality assessment and data extraction from the eligible records.

#### Extraction of primary and secondary outcome data

Change from baseline in self-reported disability and all objective measures of muscle function/impairment and functional capacity/limitation data were extracted from each study. The most frequently reported categories of secondary outcome data were selected for the secondary analysis. These were: i) measures of isometric and dynamic knee extensor strength (*KE-strength*); ii) measures from tests of *gait capacity* (i.e. max gait speed, self-selected gait speed, time to cover set distance or distance covered in set time); and iii) *lower body functional capacity* assessed by any objective test of functional capacity relying mainly on the lower body. In order to decrease outcome heterogeneity, tests including chair-rise (e.g. chair-rise tests, timed up-and-go, Short Physical Performance Battery tests) were prioritized when studies reported various functional tests.

When available, within group mean change-scores (mean_change_), baseline means (mean_base_), follow-up means (mean_end_), all corresponding measures of variability (i.e. standard deviations, SD; standard error, SE; confidence interval, CI; coefficient of variation, CV; ranges, interquartile ranges) and *p*-values of within-group change for each group were extracted. As this review focusses on the short-term effects of RT-interventions, the baseline and follow-up measures were defined as the time points nearest the initiation and termination of the interventions respectively, and the within-group mean change-score (mean_*change*_) was defined as

$$ {Mean}_{change}={Mean}_{endpoint}-{Mean}_{baseline} $$

When both the change-score including variability measure, and a complete set of baseline and follow-up means and measures of variability were missing, the authors were contacted for additional data. When the data could not be retrieved, the study was excluded from the meta-analysis, but kept in the review to be included in vote-counting analysis (supplementary conservative approach) to summarize intervention effects (described below).

#### Descriptive data

##### *Descriptive items of the included studies*

First author; year; country; setting; study design; aims of the study; participant characteristics (health status, residential status and distribution of sex); sample size in analysis; drop-out rate; compliance rate; short description of the intervention; experimental and control conditions; and the direction of the effect for the self-reported disability outcomes were extracted and complemented by relevant additional notes (displayed in Table 1). For use in a post hoc sub-analysis, participant mean age was dichotomously categorized as ≥65–79 years and ≥ 80 years respectively. Likewise, categories of gait speed at baseline (≥0.8 vs. < 0.8 m/second) was used as surrogate measure to quantify degree of functional capacity across study samples.

**Table 1:** Detailed summary of eligible studies in the review

First author, year, country	Ades et al., 2003, USA [32]	Benavent-Caballer et al., 2014, Spain [33]	Binder et al., 2002, USA [15]	Boshuizen et al., 2005, the Netherlands [34]
Setting	Cardiac rehabilitation facility	Geriatric nursing home	University indoor exercise facility	Two senior welfare centres
Design	RCT, parallel	RCT, parallel, four-armed	RCT, parallel	RCT, parallel, three-armed
Aims of the study	To evaluate the value of resistance training on measures of physical performance in older women with coronary heart disease	To evaluate the short-term effects of three different low-intensity exercise interventions on physical performance, muscle CSA and ADL.	To evaluate whether a multidimensional exercise training program can significantly reduce frailty in community-dwelling older men and women	To investigate if there are differences in the effects of an exercise intervention due to the applied intensity of supervision
Sample size (analyzed), n	IG: 19, CG: 14	IG: 22, CG: 23	IG: 66, CG: 49	IGc: 32, CG: 17
Female gender, n	Overall: 100%	IG: 68.1%, CG: 65.2%	IG: 52%, CG: 53%	IGc: 30/32 (92%), CG: 15/17 (88%)
Mean age (SD), years, range	IG: 73.2 (6.0), CG: 72.2 (5.7)	IG: 85.5 (4.7), 83.6 (5.6), 75–96	IG: 83 (4), CG: 83 (4)	IG1: 80,0 (6,7), IG2: 80,8 (5,3), CG: 77,2 (6,5) (completers only)
Participant health status (functional limitation criteria)	Patients had CHD diagnosed for > 6 months, MOS SF > 36, physical function domain score < 85	Residents in geriatric nursing home	Defined frailty criteria including: Objective test, reported ADL and IADL dependency	Difficulty in rising from a chair and unilateral knee extensor strength below 25 kgf.
Residential status	Community-dwelling	Geriatric nursing home	Community-dwelling	Apartments for elderly connected to welfare centres
Description, intensity, duration and total number of sessions	8 RT exercises focusing on leg, arm, and shoulder. Progressive program updated monthly	Low intensity RT program targeted major knee extensor muscles. 40% 1RM, 16 weeks, 48 session	Progressive whole-body RT program in weightlifting machines. 65–100% 1RM, 12 weeks, 36 sessions	9 thigh muscles exercises. Resistance provided by body weight and elastic bands. 4–8 RM (elastic band exercises) 10 weeks, 30 sessions
Control condition	Control patients met 3 times per week performing stretching, calisthenics, deep-breathing progressive-relaxation exercises, and light yoga	No intervention. Refrain from participation in exercise programs	Sham intervention: 9 flexibility exercises	No intervention. Maintain habitually active
Self-reported measure of ADL-disability/function	MOS SF-36, physical function domain	Barthel Index	Functional Status Questionnaire	The Groningen Activity Restriction Scale (ADL/IADL)
Drop-out from intervention, n	IG: 5 (21%), CG: 4 (22%)	IG: 4 (18%), CG: 4 (17%)	IG: 20 (30%), CG: 8 (16%)	IGc: 18 (36%), CG: 5 (23%)
Compliance, % (criteria)	Patients were required to attend at least 54/72 sessions (75%). 2 patients failed, and were recorded as dropouts	78% (mean attendance at sessions)	100% (attendance at sessions. Less than 100% attendance led to exclusion)	IG1: 79%, range: 57–100%, IG2: 72% range 20–93% (mean attendance at sessions)
Direction of the effect on self-reported disability/function	No effect	Positive effect	Positive effect	No effect
Adverse events	No adverse events	No adverse events	One: rotator cuff injury, and one: RT exacerbating shoulder problem	Not reported
Notes			RT is the second of three 3-months intervention phases. We consider 3- and 6-month time points as baseline and endpoint test respectively	Two eligible RT-intervention groups. Degree of supervision varied between groups
Data notes	Published and unpublished data			Data from the two intervention groups were collapsed in all analysis
Included in primary meta-analysis	Yes	Yes	Yes	Yes
First author, year, country	Buchner et al., 1997, USA [35]	Cadore et al., 2014, Spain [36]	Chandler et al., 1998, USA [16]	Chin A Paw, et al., 2006, the Netherlands [37]
Setting	Enrolees in a health maintenance organization	–	The home of the elderly	Long-term care facilities
Design	RCT, parallel, four-armed	RCT, parallel	RCT, parallel	RCT, parallel, four-armed
Aims of the study	To investigate the effect of strength and endurance training on gait, balance, physical health status, fall risk, and health service’s use in older adults	To investigate the effects of multicomponent exc. Intervention on muscle power output, muscle mass, tissue attenuation, fall risk and functional outcomes	To determine whether strength gain is associated with improvement in physical performance and disability	To evaluate the effectiveness of three different training protocols on functional performance and self-rated disabilities of older adults living in long-term care facilities.
Sample size (analyzed), n	IG: 22, CG: 29	IG: 11, CG: 13	IG: 44, CG: 43	IG: 40, CG: 31
Female gender, n	IG: 52%, CG:50%	17/24 (70%) (completers only)	Overall: 50%	IG:29/40 (73%), CG26/31 (84%)
Mean age (SD), years, range	IG: 74, CG: 75 No SD	IG: 93,4 (3,2), CG: 90,1 (1,1)	IG: 77,5 (7,1), CG 77,7 (7,8)	IG: 80,9 (5,7), CG: 81,2 (4,4)
Participant health status (functional limitation criteria)	Unable to do an 8-step tandem gait without errors, below the reference 50th percentile in KE strength	Frieds frailty criteria, institutionalized	Inability to descent stairs step by step without holding the railing	Living in long-term care facilities. The population is referred to by the authors as frail
Residential status	Community-dwelling	Institutionalized	Community-dwelling	Nursing home/residential care
Description, intensity, duration and total number of sessions	RT of the upper and lower body using Cybex Eagle weight machines. Including training at the ankle joint using adjustable weights	3 RT-exercises. 2 for knee extensors + chest press in machines (20 min). Gait and balance exercises (10 min). 8–10 RM, 12 weeks, 24 sessions	Home-based low-moderate intensity RT-programme using elastic band. Exercises target lower extremity muscles with slow velocities of movement. 10 RM, 10 weeks, 30 sessions	Long term care facility-based. 5 RT-exercises using machines, free weights and ankle/wrist weights. 60–80% 1RM, 24 weeks, 48 sessions
Control condition	Instructed to maintain usual activity	No intervention. Routine care and activities	No intervention. Controls were offered RT after the end of the trial	Attention control. Educational program led by professional creative therapist. 45–60 min twice weekly.
Self-reported measure of ADL-disability/function	Sickness Impact Profile, body care and movement subscale	Barthel Index	MOS SF-36, physical function domain	Disability in 17 ADLs
Drop-out from intervention, n	IG: 5 (20%), CG: 1 (3%)	IG: 5 (31%), CG: 3 (19%)	Overall: 13 (13%)	IG: 21 (37%), CG: 23 (45%)
Compliance, % (criteria)	IG: 95% (mean attendance at sessions)	90% (attendance at sessions. Attendance was defined as ≥90% of prescribed exercises completed)	Not reported	76% (mean attendance at sessions)
Direction of the effect on self-reported disability/function	No effect	Positive effect	No effect	No effect
Adverse events	No adverse events	Not reported	Not reported	No adverse events reported. *N* = 8 dropped out because the program was too intensive
Notes	One intervention group was eligible for inclusion in the analysis			One intervention group was eligible for inclusion in the analysis
Data notes	Ceiling effects of the Sickness Impact Profile, body care and movement-subscale was reported	Published and unpublished data	Post data not available	
Included in primary meta-analysis	Yes	Yes	No	Yes
First author, year, country	Clemson et al, 2012, Australia [38]	Danilovich et al., 2016, USA [39]	Fahlman et al., 2007, USA [40]	Hewitt et al., 2018, Australia [41]
Setting	Residents in metropolitan Sydney, Australia	Home-based, Illinois	University facilities, Urban area	Long -term residential aged care facilities
Design	RCT, parallel, three-armed	RCT, parallel	RCT, parallel, three-armed	RCT, Cluster
Aims of the study	To determine if a lifestyle integrated approach to balance and strength training is effective in reduces the rate of falls in high risk people	To test the effect of an RT-program on the physical performance and self-rated health of older adults receiving home and community-based services	To determine whether RT or a combination of RT and aerobic training resulted in the most improvement in measures of functional ability in functionally limited elders	To test the effect of published best practice exercise in long-term aged care, and determine if combined balance and progressive RT is effective in reducing the rate of falls
Sample size (analyzed), n	IG: 79, CG: 80	IG: 24, CG: 18	IG: 39, CG: 33	IG: 93, CG: 82
Female gender, n	IG: 57/105 (54,3%), CG: 58/105 (55,2%)	Overall: 83%	Not reported	IG: 71 (62.8%), CG: 73 (68.2%)
Mean age (SD), years, range	IG: 84,03 (4,38), CG: 83,47 (3,81)	CG: 74,1, CG: 75,6	IG: 74,6 (SE;1,0), CG: 76,5 (SE 1,4)	IG: 86, 65–100, CG: 86, 65–99
Participant health status (functional limitation criteria)	Two or more falls or one injurious fall in the past 12 months	Homebound, receiving long-term ADL-assistance and home management	Score < 24 on the SF-36 PFD (reference score = 30)	High- or low-care requirements (daily assistance by nurse / some assistance but not complex care-needs)
Residential status	Community-dwelling	Community-dwelling, homebound	Community-dwelling	Long-term residential care
Description, intensity, duration and total number of sessions	Structured home-based programme. 7 exercises for balance + 6 exercises for lower limb strength 3 times a week, 1 year	Health care assistant and DVD-delivered, RT program with elastic bands	RT program consisting of 13 exercises using resistive bands. Low-moderate intensity, 16 weeks, 48 sessions	Moderate intensity progressive RT program consisting of 5 exercises combined with high-progressive level balance program. 25 weeks, 50 sessions
Control condition	Sham intervention: 12 gentle flexibility exercises	No intervention. Usual care	No intervention. They were instructed to maintain their current level of activity	No intervention. Usual care
Self-reported measure of ADL-disability/function	The National Health and Nutrition Examination Surveys independence measure for Activities of Daily Living (NHANES ADL)	Patient-Reported Outcomes Measurement Information System (PROMIS), physical summary score/ADL	MOS SF-36, physical function domain	MOS SF-36, physical function domain
Drop-out from intervention, n	IG: 22 (21%), CG: 16 (15%)	IG: 3 (13%)	Not reported	IG: 16 (14%), CG: 15 (14%)
Compliance, % (criteria)	IC: 35% (SD: 29), CG: 47% (SD: 34) (adherence to programmes)	Not reported	Not reported	54% (SD: 14.3) attended at least 30 sessions (60% adherence). Median attendance: 35 sessions
Direction of the effect on self-reported disability/function	No effect	No effect	No effect	No effect
Adverse events	One Surgery for inguinal hernia due to groin strain	No adverse events	Not reported	No major events. *N* = 3 reported short-term musculoskeletal pain, *n* = 1 non-injurious fall
Notes		RT-program based on Jette 1996	One intervention group was eligible for inclusion in the analysis	48.9% of participants had a diagnosis of mild to moderate cognitive impairment
Data notes	Pre and post results are presented for different subsamples		Extraordinary small sizes of variability distorted the meta-analysis of SMDs	Pre and post results are presented for different subsamples
Included in primary meta-analysis	No	Yes	No	No
First author, year, country	Latham et al., 2003, New Zealand [42]	McMurdo and Johnstone, 1995, USA [43]	Mihalko and McAuley 1996, USA [44]	Sahin et al., 2018, Turkey [45]
Setting	Five urban hospitals in New Zealand/Australia	The home of elderly receding in sheltered housing	Nursing home or senior citizen facility	Not reported
Design	RCT, parallel, four-armed	RCT, parallel, three armed	RCT, parallel	RCT, parallel, three armed
Aims of the study	To determine the effectiveness of vitamin D and home-based quadriceps resistance exercise on reducing falls and improving physical health of frail older people after hospital discharge	To develop a low technology approach to home exercise provision for elderly people with restricted mobility	To examine the effects of upper body high-intensity strength training on muscular strength levels, ADLs, and subjective well-being in elderly males and females.	To evaluate changes in the functioning of frail older adults after undergoing RT 3 days a week for 8 weeks
Sample size (analyzed), n	IG: 112, CG: 110	IG: 21, CG: 28	IG: 29, CG: 29	IGc: 32, CG: 16
Female gender, n	IG: 55%, CG: 51%	IG: 19/21 (90%), CG: 25/28 (89%)	Overall: 83%	Not reported
Mean age (SD), years, range	IG: 80 (range: 79–81), CG: 78 (range: 77–80)	IG: 81,4 (3,4), CG:81,9 (4,7)	Overall: 82.67 (7.72)	IG1: 84.18 (6.85), IG2: 84.50 (4.81), CG: 85.37 (4.70)
Participant health status (functional limitation criteria)	Frail according to criteria (Winograd). Admitted to geriatric rehabilitation unit.	Limited mobility, dependence in ADL	19 used a wheelchair, 13 used walking assistance	Frailty according to Fried criteria
Residential status	Not specified	Sheltered housing	Nursing home	Nursing home
Description, intensity, duration and total number of sessions	Home-based quadriceps resistance program using adjustable ankle cuff weights. 3 sets of 8 reps of knee extensions in a seated position.	Low technology, low cost home exercise program using elastic bands. Emphasis on safety and respect for pain. 6 months with training on daily basis. No data on intensity	Upper body RT program with one exercise for the following muscle-groups: pectorals, latissimus dorsi, deltoids, biceps, and triceps. Performed with dumbbells	11 RT exercises for upper and lower body. 1 set of 6–10 reps at a slow speed (6–8 s/rep). IG1: 70% 1RM IG2: 40% 1RM. 8 weeks, 24 sessions
Control condition	Received frequency-matched telephone calls and home visits from physical therapist who inquired about patient’s recovery, gave general advice.	Frequency and duration matched health education program. Informal discussions on exercise, diet, sleep, meditation, stress foot care and safety	Upper body, no-stress exercise program: Breathing techniques; movement of the neck, shoulder, arms, hands, and torso; and mild stretching activities	Instructed to continue usual daily routines
Self-reported measure of ADL-disability/function	MOS SF-36, physical function domain	Barthel Index	Barthel Index, tailored	Barthel Index
Drop-out from intervention, n	IG: 8 (7%), CG: 13 (10%)	Overall: 20%	Not reported	IGc: 0, CG: 0
Compliance, % (criteria)	82% (mean attendance at sessions)	Not reported	Not reported	Not reported
Direction of the effect on self-reported disability/function	No effect	No effect	Positive effect	Positive effect
Adverse events	The exercise group had an increased risk of musculoskeletal injury and higher scores of fatigue.	No adverse events	Not reported	Not reported
Notes		One intervention group was eligible for inclusion in the analysis		Two eligible RT-intervention groups. Work load intensity varied between groups.
Data notes	Missing baseline data		ANCOVA test applied to account for baseline imbalances	Data from the two intervention groups were collapsed in all analysis but the sub-analysis for training intensity
Included in primary meta-analysis	No	Yes	Yes	Yes
First author, year, country	Seyennes et al., 2004, France [17]	Timonen et al., 2006, Finland [46]	Venturelli et al., 2010, Italy [47]	Westhoff et al., 2000, the Netherlands [18]
Setting	Public nursing homes	Primary care health centre	Geriatric institute	Home-based/community centre-based
Design	RCT, parallel, three-armed	RCT, parallel	RCT, parallel	RCT, parallel
Aims of the study	To measure dose-response effect of a free weight-based RT program on KE muscle function, functional limitation and self-reported disability.	To determine the effects of a group-based exercise program on ADL and IADL activities relevant to daily life after discharge from hospital	To evaluate the feasibility of upper-body circuit-RT program, and to verify if arm training improves physical outcomes, ADL-function and cognitive outcomes.	To investigate if a 10-week low-intensity strength training program can improve strength of the knee extensors and functional ability in frail elderly.
Sample size (analyzed), n	IGc: 14, CG: 8	IG: 26, CG: 30	IG: 12, CG: 11	IG: 10, CG: 11
Female gender, n	Not reported	IG: 100%, CG: 100%	IG: 100%, CG: 100%	Not reported
Mean age (SD), years, range	IG1: 83.3 (2.8), IG2: 80.7 (2.3), CG: 80.3 (2.0)	IG: 83.5 (4.1) CG: 82.6 (3.7)	IG: 83,3 (6,7), CG: 84,1 (5,8)	IG: 75.9 (6.8), CG: 77.5 (8.1)
Participant health status (functional limitation criteria)	Institutionalized. Characterised by authors as frail. Objective measure not reported	Hospitalized due to an acute illness and mobility-impaired	Dependent in one or more ADL (BI), serious mobility limitation, MMSE > 15 < 25	Difficulty in rising from a chair
Residential status	Public nursing home	Community-dwelling	Geriatric institute	Residents of assistant living facilities
Description, intensity, duration and total number of sessions	Classical progressive RT of the KE muscles using ankle cuffs. IG1: 80% 1RM, IG2: 40% 1RM, 10 weeks, 30 sessions	Group based progressive RT with weight training equipment plus functional exercises. 8–10 RM, 10 weeks, 20 sessions	Group based upper body RT program using dumbbells, looped, elastic bands, sticks and sponge balls. Progression by raising number of repetitions and or load	Individually tailored RT program for the KE using bodyweight and elastic band to provide resistance. 9 exercises. 4 RM (elastic band exercises), 10 weeks, 30 sessions
Control condition	Placebo: similar program with empty ankle cuffs	Instructions for a home exercise training program, including functional exercises. No further encouragement to exercise.	Kept their habits unaltered throughout the study. Were provided physiotherapy as usual	No intervention. Asked to continue with their normal activities
Self-reported measure of ADL-disability/function	Health Assessment Questionnaire	Tailored ADL/IADL function scale	Barthel Index	The Groningen Activity Restriction Scale (ADL/IADL), lower extremity-specific domain
Drop-out from intervention, n	Overall: 5 (19%)	IG: 8 (23%), CG: 4 (12%)	IG: 3 (20%), CG: 4 (27%)	IG: 4 (29%), CG: 1 (8%)
Compliance, % (criteria)	99% (criteria not stated)	90%, range 55–100% (mean attendance at sessions)	75% (SD: 16%) (mean attendance to sessions)	87% (mean attendance to sessions)
Direction of the effect on self-reported disability/function	No effect	No effect	Positive effect	Positive effect
Adverse events	No adverse events	Not reported	No adverse events	No adverse events
Notes	Two eligible RT-intervention groups. Work load intensity varied between groups. 5 drop outs in total. Number of dropouts on group-level is not reported.	ADL/IADL measured by proxy (health care personnel)	Very frail subjects - many are wheelchair users	
Data notes	Published and unpublished data. Data from two intervention groups were collapsed in all analysis but the sub-analysis for training intensity	Data not suitable for meta-analysis		
Included in primary meta-analysis	Yes	No	Yes	Yes

*ADL* activities of daily living, *CG* control group, *CHD* coronary heart disease, *IADL* instrumental activities of daily living, *IG* intervention group, *KE* knee extensors, *MMSE* mini-mental state examination, *MOS SF-36* Medical Outcomes Study 36-Item Short Form Health Survey, *PFD* physical function domain, *RCT* randomized controlled trial, *RM* repetition maximum, *RT* resistance training, *SD* standard deviation

##### RT-intervention items

In order to describe the extent of heterogeneity in the included RT-programs, data regarding: i) training intensity; ii) duration; iii) frequency; iv) supervision; and v) progression protocols were extracted. A previous review [12] found that training intensity and duration were significant predictors of the effect of RT on muscle strength. Therefore, these two variables were selected as covariates in two independent meta-regressions in order to investigate whether, and to what extent the two variables predict self-reported ADL-disability. For training intensity, percentage of one Repetition-Maximum (%1RM) was selected as the standardized unit and used as a continuous covariate for the meta-regression. For studies presenting intensity as number of RMs (nRM, i.e. the maximal number of repetitions with a given load) this was translated into %1RM using Brzycki’s equation [48]. When a range of intensities was provided, the mean intensity rounded up to nearest 5% as the estimate was used. Intensities based on Rate of Perceived Exertion scales (RPE) did not form part of the covariate.

### Quality assessment/risk of bias in individual studies

Risk of bias in individual studies was evaluated using the validated PEDro scale [28, 49]. The PEDro quality assessment tool rates the internal validity of RCTs on a scale from 0 (low quality) to 10 (high quality), with a score of ≥6 representing a cut-off for high-quality studies. For this review, two modifications to the scale were made. When awarding points in item four: *“the groups were similar at baseline regarding the most important prognostic indicators”* original texts and tables were screened for evidence that baseline differences were assessed. However, equality in baseline levels regarding self-reported disability was not always investigated, as this was a secondary outcome in many studies. Consequently, we performed meta-analysis of baseline-scores to screen for potential baseline differences in self-reported disability that were not addressed in the original articles [50].When this analysis revealed a baseline value in the active intervention group that differed significantly from that of the control group in a study, that study was awarded a “no” in item four of the PEDro scale, regardless of whether this baseline difference was addressed in the original paper.True participant blinding by a placebo intervention is not possible given the nature of the active treatment (i.e. RT). Item 5 *“there was blinding of all participants”* was therefore considered satisfied if sham intervention or attention control was applied in the control group.

### Summary measures

Due to the various eligible self-reported ADL-disability scales, the main summary measure was the standardized mean difference (SMD) in change-scores and the corresponding confidence interval (CI). The size of the pooled SMD was interpreted according to the following rule of thumb < 0.40 = small effect, 0.40 to 0.70 = moderate effect, > 0.70 = large effect ([51]; ch. 17.8.2).

### Handling of missing data

Missing SDs were imputed from other available measures of variability (SE, CI) or from the exact *p*-values using methods proposed by Fu et al. [50]. When only a baseline mean, a follow-up mean and the corresponding measures of variability were reported, the mean_change_ was calculated based on these data whereas SD_change_ was imputed from correlation estimates (Corrs) from other studies using the following eq. ([50, 51]; ch. 16.1.3.2).

$$ {SD}_{change}=\sqrt{SD_{baseline}^2+{SD}_{endpoint}^2-\left(2\times {Corr}_{mean}\times {SD}_{baseline}^2\times {SD}_{endpoint}^2\right)} $$

Corr_mean_ is the mean of all Corrs that could be calculated for the given category of outcome (i.e. self-reported, KE-strength, lower body physical function, or gait capacity). The Corrs for the individual studies were calculated by the below equation when studies provided sufficient information.

$$ Corr=\frac{SD_{baseline}^2+{SD}_{endpoint}^2-{SD}_{change\ score}^2}{2\times {SD}_{baseline}^2\times {SD}_{endpoint}^2} $$

If very few studies provided the data needed to calculate the Corr_mean_, missing SD’s were imputed directly from the other treatment group within the same study or from another included study. Where studies reported multiple intervention groups of more than one RT modality (i.e. varying level of intensity, supervision or frequency) versus a control condition, data were combined according to existing recommendations ([51]; ch. 7.7.3.8 and 16.5.4). Non-parametric summaries were used to estimate means and SDs in two studies [39, 43] regardless of skewed distribution. This approach is supported by Fu et al. [50] provided the variable of interest has symmetric distribution in most included studies, as was the case in this meta-analysis. An exact description of how missing data have been handled for each study can be retrieved from the corresponding author.

### Synthesis of results

The data synthesis was carried out using Stata statistical software (Stata/IC 15.1). The results from the individual studies were combined and pooled by calculating adjusted SMDs using Hedges’ g. Accordingly, meta-analyses were performed for the primary and the secondary outcomes. For scales where low scores are favourable, the means were multiplied by − 1. Considering the broad inclusion criteria for the resistance training interventions, true heterogeneity in intervention effects was expected and the DerSimonian-Laired random-effects method for continuous outcomes was applied accordingly. The extent of between study heterogeneity was tested with the standard Q^2^ statistics and the *I*^2^ index [52]. There is somewhat agreement across references [51]; ch. 9.5.2, [53]) that heterogeneity should be assumed if *I*^2^ is > 50%, indicating that 50% of the variability in the outcome cannot be explained by sampling variation, and cut points of I^2^ values of 25, 50, and 75% may be used to categorize low, moderate, and high amounts of heterogeneity [54].

### Secondary analysis

To investigate if the intervention effects on objective measures of functioning were also associated with the changes in self-reported ADL-disability, meta-regressions were performed as follows: the effect sizes (i.e. SMDs) calculated in the meta-analyses on KE-strength, gait capacity and lower body functional capacity were fitted as continuous covariates in three separate meta-regressions (metareg-command) using the effect size (i.e. SMD) on self-reported disability/function as the dependant variable and the standard error of that SMD to weight the studies. Three measures from the meta-regressions were used to interpret the results [55]: i) the *I*^*2*^_*res*_*%* is the percentage of the residual variation that is due to between-study heterogeneity (the rest of the heterogeneity (100% - I^2^_res_%) is due to within-study sampling variability); ii) the *adj. R*^*2*^*%* which is the proportion of the heterogeneity in the dependent factor that can be explained by the covariate fit in the meta-regression, and iii) a *p*-value of the overall test of the covariate in the random effects model.

In addition, the predictive value of specific intervention parameters (duration of intervention and load intensity*)* on the size of the RT effect on self-reported disability/function were tested by meta-regression as described in the section above [55]. Heterogeneity sources were also investigated by performing stratified analyses according to participant age, (65 < 80 yr., ≥80 yr.), residential status and relevant study quality parameters (parameters selected post hoc) [53].

### Risk of publication bias across studies

To assess whether publication bias influenced the results of the primary outcome, a funnel plot was created [56] and the Egger’s test [57] (*metabias* command in Stata) was applied to assess small study effect.

## Results

### Search and study selection

**Fig. 1:**
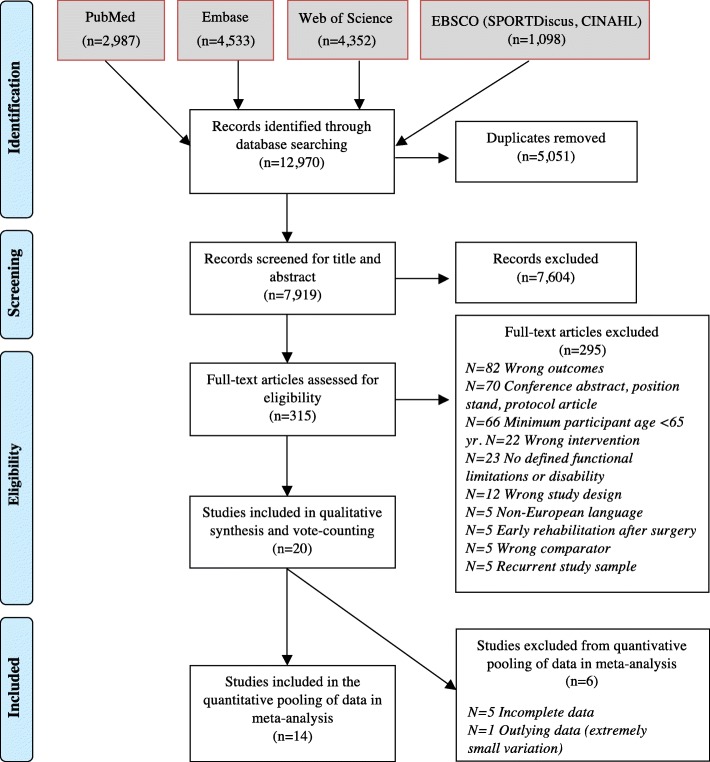
PRISMA flow chart of the study selection process

The result of the search is outlined in the PRISMA diagram (Fig. 1). The search yielded 12,970 records, of which 5051 were duplicates. Thus, the title/abstract of 7919 records were screened for possible eligibility, leading to the exclusion of 7604 records based on the inclusion and exclusion criteria. The full text of 315 records were assessed for eligibility and 295 records were excluded (Fig. 1). Of the 20 eligible records, 14 included complete data that enabled their inclusion in the primary meta-analysis. In five of the remaining six records, the relevant data were incomplete and was not provided upon request [16, 38, 41, 42, 46]. The sixth trial [40] was initially included in the main meta-analysis. However, exceptionally small sizes of variation in change in this study, heavily distorted the results of the meta-analysis (forest-plot of the meta-analysis including this study is included in Additional file 2, Fig. 1a). Consequently, it was decided to exclude the data from this study from the quantitative pooling. Not taking these six studies into account could however increase the risk of systematic, selective reporting potentially leading to an overly positive conclusion. Consequently, we made a post hoc decision to keep the six trials in the qualitative synthesis of data and include them in an additional vote-counting analysis * ([51]; ch. 9.4.11, [58]). The vote-counting procedure involves simply comparing the number of studies reporting positive effect (intervention favours experimental group), no effect (the effect was insignificant) and negative effect (intervention favours control group). If a majority of studies fell into any of these three categories, this category was declared the best estimate of the direction of the true relationship between the independent variable (i.e. RT-intervention) and the dependent variable (i.e. self-reported ADL) [58]. This method has major limitations as it does not take into account the quality of the studies, the size of the samples or the size and variability of the effect. Bushman & Wang [58] advocate that this method should be used only as a supplementary data synthesis approach to complement the primary meta-analysis of SMDs, as was the case in this study.

### Study characteristics

A detailed description of the characteristics of the eligible studies is presented in Table 1.

Overall, a total of 1422 participants were included in the 20 RCTs, with 747 and 675 participants in the intervention and control groups respectively. In the subsample of trials included in the primary meta-analysis (*n* = 14), the total number of participants was 651(IG: *n* = 354, CG: *n* = 297). Sample size ranged from 21 to 222 participants (median/mean 50/71). In three trials, the mean age of participants was under 75 years [32, 35, 39], all of which were included in the meta-analysis. Eight trials recruited participants who were community-dwelling [15, 16, 32, 35, 38–40, 46], eight trials recruited institutionalized elderly [17, 33, 36, 37, 41, 44, 45, 47], three trials recruited from sheltered housing [18, 34, 43], and one trial did not specify residential status [42]. In the five trials that were not included in the main meta-analysis, the participant mean age was above 75 years and they were not institutionalized. The distribution of participant sex was unequal with females being more represented than males. Three included only women [32, 46, 47], eight trials had > 60% of female participants [33, 34, 36, 37, 39, 41, 43, 44], five trials included men and women nearly evenly (50–60% female) [15, 16, 35, 38, 42] and four studies did not report sex distribution [17, 18, 40, 45]. Three trials [17, 34, 59] had two eligible RT-intervention groups for which data were collapsed as described previously. Regarding the comparison condition, nine trials provided a sham intervention as stretching or exercises without load or attention control equalling or approximating the time spent in RT-intervention [15, 17, 32, 37, 38, 42–44, 46]. The control groups in the remaining 11 studies were asked to maintain their current activity level and were provided with usual care when relevant. Four studies did not report compliance [16, 40, 43, 44]. Compliance in the remaining 16 studies was relatively high with mean compliance rates at ~ 75% or above. Compliance was in most cases expressed as $\frac{\mathrm{n}\ \mathrm{attended}\ \mathrm{sessions}\ }{\mathrm{n}\ \mathrm{planned}\ \mathrm{sessions}} \times 100\%$. In one study however, a specific compliance criterion was set and compliance below this threshold was characterised as drop-out, making 100% compliance inevitable [32]. All but two studies [40, 44] reported drop-out. The mean drop-out rate was higher among IG’s compared to CG’s (IG mean drop out: 20%, range 6 to 37% vs. CG mean drop out: 15%, range 0 to 45%). One study reported participants dropping out for reasons related to the intervention (e.g. exercise intensity [37]). Seven studies reported some type of adverse events. Major adverse events were reported in two of these studies, with one case of rotator cuff injury [15] and one inguinal hernia [19]. Muscle injury and occasional exacerbation of musculoskeletal conditions such as arthrosis were the most frequently reported adverse events [15, 41, 42]. In six studies, no data on adverse events was presented [16, 34, 40, 44–46].

#### Resistance training programs

For detailed descriptive overview of the identified RT programs see Table 2. Overall, the majority of exercise programs were characterized as supervised (*n* = 18), having a progression protocol (*n* = 17) and being well described (i.e. exercise equipment or strategy for applying external resistance was reported, *n* = 20). The RT exercise programs were home-based in 7 trials [16, 18, 34, 38, 39, 42, 43] (including two partly facility-based trials [18, 34]). One home-based trial was completely unsupervised [38] and one trial failed to report supervision status [40]. The level and type of supervision varied among the 20 included studies. Supervision frequencies included once monthly [43], once weekly [42], two out of every three sessions [18, 34] and each session [15, 17, 32, 33, 35–37, 39, 44, 46, 47]. In one trial [39], the usual care staff undertook the training supervision and one study provided guidance by videotape [37]. Exercise progression was not described in three studies [43, 45, 46]. Elastic bands, exercise machines, ankle cuff weights and free weights were nearly evenly being used (elastic bands *n* = 7 [16, 18, 34, 39, 40, 43, 47]; machines *n* = 6 [15, 35–37, 41, 46]; ankle cuff weights *n* = 6 [17, 33, 37, 38, 42, 45]); free weights *n* = 5 [15, 32, 37, 45, 47]). Use of body weight in addition to an external resistance was reported in three trials [16, 18, 34]. Sixteen trials [15, 16, 18, 32, 34–45] reported the number of key RT exercises (median = 8, range 1–24). The median program duration was 12 weeks, ranging from eight to 52 weeks. The most common exercise frequency was three times a week (15 trials [15–18, 32–35, 38–40, 42, 44, 45, 47]) followed by twice a week (4 trials [36, 37, 41, 46]). One home-based trial [43] encouraged the participants to perform the RT program on a daily basis resulting in the highest number of prescribed sessions (168 sessions). The median number of prescribed sessions was 36 and the lowest number was 20. The number of sets per exercise varied from one to three, most commonly comprising 8 to 12 repetitions per set (ranging from four to 20 repetitions per set). The calculation of training intensity was based on different strategies among the studies. The most frequently used strategy was to perform a formal RM test (9 trials [15, 17, 32, 33, 35, 36, 42, 44, 47]). Six studies failed to report any strategy for calculating the targeted load intensity [16, 37–39, 45, 46]. RPE was used as the only strategy to set the intensity level in two studies [40, 41], and in combination with RM in another study [32]. The applied workload intensities were accordingly reported in a number of ways, most regularly as %1RM (10 trials [15, 17, 32, 33, 35–37, 42, 45, 47]), followed by nRM (5 trials [16, 18, 34, 44, 46]), and RPE (3 trials [39–41]). Two studies did not report workload intensity [38, 43]. Estimation of within-study mean %1RM could be obtained as described in the method section in 15 trials [15–18, 32–37, 42, 44–47] and the estimated mean intensity was ~ 70%, ranging from 40 to 85%. Eight of the fifteen trials applied mean intensities at 70% 1RM and above [15–18, 32, 34, 45, 46] with two studies having an additional low intensity-intervention group [17, 45].

**Table 2:** Detailed overview of resistance training programs in the eligible studies

	Equipment/external load	Home(H) or Facility-based (F) individual (I) or Group-based (G)	number of RT exercises	Specific muscle groups/lower body/upper body/whole body	Progression protocol	Intensity was based on RM test protocol	Intensity was based on RPE	Supervised by instructor	Training period (weeks)	Training frequency (sessions/week)	Prescribed training volume, (n sessions total)	Session duration in minutes. Total time (incl. Warm-up) / RT time only	Number of sets	Number of repetitions per set	Load intensity (LI)	Estimated LI (%1RM)€
Ades et al., 2003 [32]	Weights/ dumbbells	F/ND	8	Whole body	Yes	Yes	Yes	Yes	24	3	72	ND	1–2	10	80% 1RM	80
Benavent-Caballer et al., 2014 [33]	Ankle cuffs	F/I	ND	KE	Yes	Yes	No	Yes	16	3	48	30–35 / -	3	15	40% 1RM	40
Binder et al., 2002 [15]	Free weights/ machines	F/G	6	Whole body	Yes	Yes	No	Yes	12	3	36	ND	3	8–12	65–100% 1RM	82.5
Boshuizen et al., 2005 [34]	Body weight/ elastic bands	H + F/G	9	Lower body	Yes#	No	No	Yes***	10	3	30	ND	3	4–8	4–8 RM (elastic band exercises)	85
Buchner et al., 1997 [35]	Machines	F/G	9	Whole body	No	Yes	No	Yes	24	3	72	60 / 35–45	2	10	1st set: 50–60% 1RM. 2nd set: 75% 1RM	62.5
Cadore et al., 2014 [36]	Machines	F/ND	3	KE + chest	Yes	Yes	No	Yes	12	2	24	40 / 20	ND	8–10	40–60% 1 RM	50
Chandler et al., 1998 [16]	Body weight/ elastic bands	H/I	8	Lower body	Yes	No	No	Yes	10	3	30	ND	2	10	10 RM (elastic band)	75
Chin a Pow et al., 2006 [37]	Machines/ free weights/cuff weights	F/G	5	Whole Body	Yes	No	No	Yes*	24	2	48	45–60 / -	2	12	60–80% 1RM	70
Clemson et al., 2012 [38]	Ankle cuffs	H/I	6	Lower body	Yes	No	No	No	52	3	156	ND	ND	ND	ND	ND
Danilovich et al., 2016 [39]	Elastic bands	H/I	11	Whole body	Yes	No	No	Yes**	12	3	36	35 / 25	1	max 10	“Moderate”	low/ mod
Fahlman et al., 2007 [40]	Elastic bands	ND/ND	13	Whole body	Yes	No	Yes	No	16	3	48	ND	2	12	To mild fatigue §	low/ mod
Hewitt et al., 2018 [41]	Pneumatic machines	F/G	5	Whole body	Yes	No	Yes	Yes	25	2	50	60 / -	3	10	12–14(Borg)	mod
Latham et al., 2003 [42]	Ankle cuffs	H/I	1	KE	Yes	Yes	No	Once weekly	10	3	30	ND	3	8	50% 1RM	50
McMurdo & Johnstone 1995 [43]	Elastic bands	H/I	24	Whole body	No	No	No	once monthly	24	7	168	15 / -	1	5–10	ND	ND
Mihalko & McAuley 1996 [44]	Dumbbells	F/ND	5	Upper body	Yes	Yes	No	Yes	8	3	24	30 / -	ND	10–12	10–12 RM	70
Sahin et al., 2018 [45]	Ankle cuffs, dumbbells	ND/ND	11	Whole body	No	No	No	Yes	8	3	24	40 / 20	1	6–10	IG1: 70% 1RM, IG2: 40% 1RM	40,70
Seynnes et al., 2004 [17]	Ankle cuffs	ND/ND	ND	KE	Yes	Yes	No	Yes	10	3	30	ND	3	8	IG1: 80% 1RM, IG2: 40% 1RM	40, 80
Timonen et al., 2006 [46]	Machines	F/G	ND	Lower body	No	No	No	Yes	10	2	20	90 / 30	2	8–10	8–10 RM	80
Venturelli 2010 [47]	Elastic bands/ barbells	F/G	ND	Upper body	Yes	Yes	No	Yes	12	3	36	45 / 25	3	20	50% 1RM	50
Westhoff et al., 2000 [18]	Body weight/ elastic bands	F + H/ND	9	KE	Yes #	No	No	Yes***	10	3	30	60 / 40	1–3	4–8	4–8 RM (elastic band)	85

*RT* Resistance Training, *LI* Load Intensity, *RM* Repetition Maximum, *mod* moderate intensity, *Borg* BORG Rate of Perceived Exertion scale, *KE* Knee Extensors, *ND* No Data/not reported

#### Primary outcome - self-reported ADL-function/disability

A full list of all self-reported ADL outcomes extracted from the articles is presented in the additional material (Additional file 2, Table 1A). In total, 10 different instruments for self- or proxy rating of function/disability were represented in the 20 included studies, of these nine were represented in the primary meta-analysis. All instruments were generic (i.e. not condition-specific). The physical function domain (PFD) of the SF-36 and the Barthel Index (BI) [60] were the most frequent outcomes, used in five trials each (SF-36 [16, 32, 40–42]; BI [33, 36, 43, 45, 47]). However, the SF-36 was only represented in the meta-analysis by one study [32]. Different scoring systems were applied across studies for both of these two instruments. The Groningen Activity Restriction Scale was applied in two studies [18, 34], one of them presenting separate data on lower extremity-related ADL, which was the target body part of that study intervention [18]. Lowton and Brody’s IADL Scale was likewise used in two studies, but not in its full version. Six identified instruments were only applied in one study each [15, 17, 22, 35, 37, 39, 44, 46]. Four instruments were referred to by the study authors as validated, supported by a reference (the SF-36 [32, 40], the BI [36], the Groningen Activity Restriction Scale [18, 34], the Joensuu classification of ADL/IADL skills [46]).

#### Secondary outcomes - objective study outcomes

Objective study outcomes were only collected from the trials that were included in the main meta-analysis (*n* = 14). KE-strength was reported in nine studies [15, 17, 18, 34–37, 39, 45]. Eligible data for the lower body functional capacity were available from 12 studies [15, 17, 18, 32–37, 39, 43, 45]. Chair-rise as a single task or chair-rise in combination with walking and/or balance were the most frequent outcomes in this covariate [17, 18, 33, 34, 36, 37, 39, 43, 45]. Two studies [15, 32] used a battery of functional tests for the entire body. However, one of them [32] reported a separate score for the tests mainly related to the lower body, but it was not specified in the text which tests were selected for this score. Nine studies presented data from gait performance tests [18, 32–37, 39]. Three trials measured the distance reached in six minutes of walking [17, 32, 33], and the remaining six trials measured time to complete a pre-set short walking distance [18, 34–37, 39]. A descriptive overview of secondary outcomes is available in the additional material (Additional file 2, Table 2A).

### Risk of bias within studies

#### Rating of methodological quality

A full overview of the assessment of study quality by the PEDro tool is summarized in Table 3. The majority of studies (15 out of 20) were of high quality. Nine trials [15, 16, 18, 36–39, 41, 43] provided some information about the method of randomization, suggesting that randomization was properly concealed (i.e. the use of concealed envelopes or the randomization was generated by an independent person). Consequently, this item was the least frequently met in the studies. Baseline imbalance between groups were reported in two studies [44, 47] who accounted for this by using analysis of covariance (i.e. ANCOVA). The meta-analysis of baseline data revealed baseline differences in self-reported ADL-disability outcome in further two studies [18, 33]. The quality criterion *“8: Measures of at least one key outcome was obtained from 85% of the subject initially allocated to groups”*, was only fulfilled by 10 trials [15–17, 33, 35, 39, 41, 42, 44, 45], death and severe illness unrelated to the intervention were often reported as major dropout reasons.

**Table 3:** Asessment of study quality by the PEDro Scale

	Ades et al., 2003^a^ [32]	Benavent-Caballer et al., 2014^a^ [33]	Binder et al., 2002^a^ [15]	Boshuizen et al., 2005^a^ [34]	Buchner et al., 1997^a^ [35]	Cadore et al., 2014^a^ [36]	Chandler et al., 1998^b^ [16]	Chin a Pow et al., 2006^a^ [37]	Clemson et al., 2012^b^ [38]	Danilovich et al., 2016 ^a^ [39]	Fahlman et al., 2007^b^ [40]	Hewitt et al., 2018^b^ [41]	Latham et al., 2003^b^ [42]	McMurdo & Johnstone 1995^a^ [43]	Mihalko & McAuley 1996^a^ [44]	Sahin et al., 2018^a^ [45]	Seynnes et al., 2004^a^ [17]	Timonen et al., 2006^b^ [46]	Venturelli et al., 2010^a^ [47]	Westhoff et al., 2000^a^ [18]
1. Eligibility criteria were specified (external validity)	Y	Y	Y	Y	Y	Y	Y	Y	Y	Y	Y	Y	Y	Y	Y	Y	Y	Y	Y	Y
2. subjects were randomly allocated to groups (in a crossover study, subjects were randomly allocated an order in which treatments were received)	Y	Y	Y	Y	Y	Y	Y	Y	Y	Y	Y	Y^c^	Y	Y	Y	Y	Y	Y	Y	Y
3. Allocation was concealed	N	N	Y	N	N	Y	Y	Y	Y	Y	N	Y	N	Y	N	N	N	N	N	Y
4. The groups were similar at baseline regarding the most important prognostic indicators	Y	N^a^	Y	Y	Y	Y	Y	Y	Y	Y	N	N^b^	Y	Y	N^b^	Y	Y	Y	N^b^	Y
5. There was blinding of all subjects	Y	N	Y	N	N	N	N	Y	Y	N	N	N	Y	Y	Y	Y	Y	Y	N	N
6. There was blinding of all therapists who administered the therapy	N	N	N	N	N	N	N	N	N	N	N	N	N	N	N	N	N	N	N	N
7. There was blinding of all assessors who measured at least one key outcome	N	Y	Y	Y	Y	Y	Y	Y	Y	Y	N	Y	Y	Y	N	N	Y	N	Y	Y
8. Measures of at least one key outcome were obtained from more than 85% of the subjects initially allocated to groups	N	Y	Y	N	Y	N	Y	N	N	Y	N	Y	Y	N	Y	Y	Y	N	N	N
9. All subjects for whom outcome measures were available received the treatment or control condition as allocated or, where this was not the case, data for at least one key outcome was analysed by “intention-to-treat”	N	Y	N	N	Y	Y	N	N	Y	Y	N	Y	Y	Y	Y	Y	N	N	Y	N
10. The results of between-group statistical comparisons are reported for at least one key outcome	Y	Y	Y	Y	Y	Y	Y	Y	Y	Y	Y	Y	Y	Y	Y	Y	Y	Y	Y	Y
11. The study provides both point measures and measures of variability for at least one key outcome	Y	Y	Y	Y	Y	Y	Y	Y	Y	Y	Y	Y	Y	Y	Y	Y	Y	Y	Y	Y
Total score for internal validity (item 2–15)	5	6	8	5	7	7	7	7	8	8	3	6	8	8	6	6	7	5	5	6

For more information on the PEDro scale see Elkins et al., 2010 [28]

Y = yes, N = no, a = study included in primary meta-analysis and vote-count analysis, b = study only included in vote-count analysis

^a^Baseline imbalance in self-reported ADL outcome is based on meta-analysis of pre-scores^b^ Baseline imbalance was accounted for in the statistics

^c^Cluster randomization

### Risk of bias across studies

#### Publication bias

**Fig. 2:**
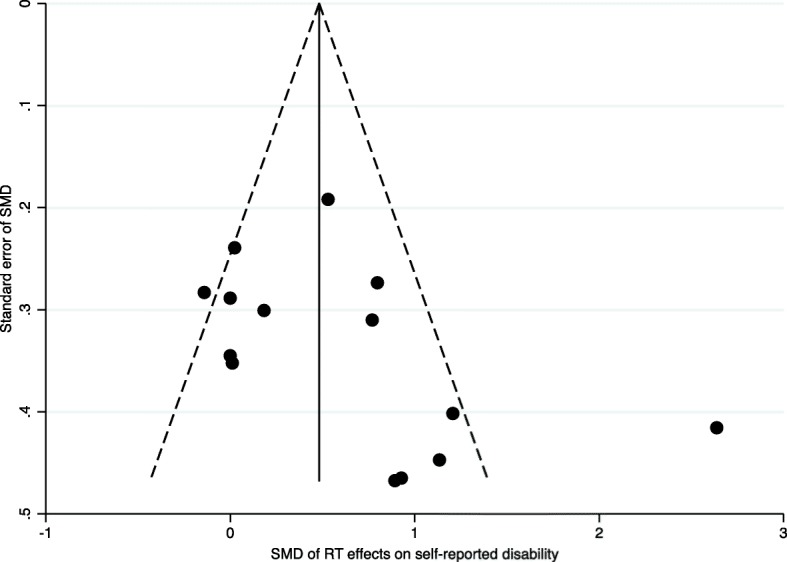
Funnel plot with pseudo 95% confidence limits. Funnel plot illustrating risk of publication bias in the analysis of effect of resistance training interventions on self-reported disability. SMD = standardized mean difference

The Egger’s test for small study effects was not significant (*p* = 0.05), but visual inspection of the funnel plot showed one outlier [45] (Fig. 2). When removing that outlier from the dataset there was no indication of publication bias (Symmetric funnel plot and Eggers test, *p* = 0.11). The outlying study [45] was of high quality according to the PEDro score (score = 7).

### Results of individual studies and synthesis of results

#### Primary analysis - pooled effects of RT on self-reported disability

**Fig. 3:**
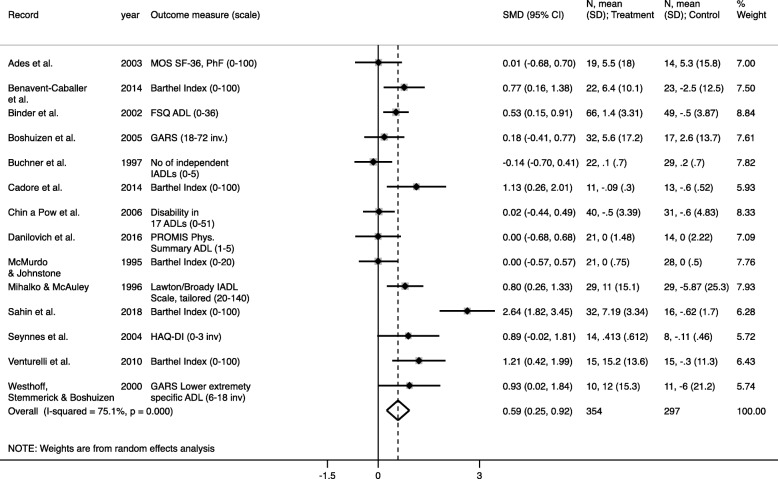
Effects of resistance training on measures of self-reported disability/function. Forest plot from meta-analysis of the effect of resistance training interventions on self-reported disability or function in older adults. Results are from random effects model using Hedges’ g. SMD = standardised mean difference; N = number of participants in the group; SD = Standard Deviation; ADL = Activities of Daily Living; IADL = Instrumental Activities of Daily Living; MOS SF-36 = The Medical Outcome Study 36-item Short Form Survey; GARS = the Groningen Activity Restriction Scale (ADL/IADL); FSQ = Functional Status Questionnaire; HAQ-DI=Health Assessment Questionnaire

Data from 14 trials were included in the primary meta-analysis (Fig. 3, Table 4). When the SMDs from these trials were pooled using a random effects model, a significant moderate positive effect of RT was found (SMD: 0.59 95% CI: 0.253 to 0.925, z = 3.43, *p* = 0.001) (Fig. 3). However, between-study heterogeneity was high (I^2^ statistic = 75,1%, Q = 52.26 on 13 degrees of freedom (d.f.), *p* <  0.001) (Table 4).

**Table 4:** Results from primary meta-analysis on self-reported disability and secondary meta-analysis on objective measures of physical function and muscle strength

	Summary statistics					Heterogeneity statistics			
	N	SMD	95% CI low	95% CI high	p-value	Q-stat	d.f	Q-stat. *p*-value	I^2^%
Effect of RT on self-reported disability	14	0.589	0.253	0.925	0.001	52.26	13	< 0.001	75.1
Effect of RT on gait capacity	9	0.36	−0.016	0.73	0.061	21.96	8	0.005	63.6
Effect of RT on lower body functional capacity	12	0.625	0.223	1.026	0.002	54.42	11	< 0.001	79.8
Effect of RT on knee extensor strength	9	0.970	0.456	1.485	< 0.001	48.22	8	< 0.001	83.4

*RT* resistance training, *SMD* standardized mean difference

#### Complementary analysis – vote-count procedure

All eligible trials (*n* = 20) were included in the secondary analysis of vote-counts. Seven trials demonstrated a significant effect of RT on self-reported disability, while no trials found effects favouring the control group. Thus, *no effect of RT on self-reported disability* was the most frequent finding among the studies in this review (*n* = 13 studies). None of the six trials, that were ineligible for the meta-analysis, found a significant intervention effect on self-reported disability (Table 1).

### Secondary analysis

#### Investigation of between-study heterogeneity

The apparent statistical heterogeneity in the pooled data (I^2^ = 75.1%; Q: *p* < 0.001) was explored using sub-group analysis according to three participant characteristics (age; residential status; and gait speed at baseline), two training modality parameters (workload intensity and session frequency) and six study-quality items (allocation concealment, baseline imbalance, subject blinding, assessor blinding, end point data on 85% of participants, and intention-to-treat analysis). The latter were selected post-hoc because they were not satisfied in all trials (see Table 3).

All of these subgroup analyses resulted in either non-significant pooled SMDs, heterogeneity, or a very low number of trials (< 4) within the new subgroups (see results in Table 5).

**Table 5:** Results from stratified meta-analyses on the effects of resistance training on self-reported disability/function in older adults with functional limitation or disability

	Summary statistics							Heterogeneity statistics			
				95% CI	95% CI	Weight				Q-stat.	
Variable	Sub-group	N	SMD	low	high	%	*p*-value	Q-stat	d.f	*p*-value	I^2^%
Overall effect of RT on self-reported disability		14	0.589	0.253	0.925	100.00	0.001	52.26	13	< 0.001	75.1
Participant characteristics											
Participant mean age	< 80 years	5	0.106	−0.192	0.404	35.3	0.485	4.12	4	0.390	2.9
	≥80 years	9	0.830	0.381	1.280	64.7	0.001	38.99	8	< 0.001	79.5
Residential status	CD	4	0.163	−0.194	0.520	30.8	0.370	5.00	3	0.172	40.0
	SH	3	0.262	−0.199	0.723	21.1	0.265	2.91	2	0.233	31.1
	GI	7	1.027	0.438	1.617	48.1	0.001	31.67	6	< 0.001	81.1
Gait speed at baseline	≥ 0.8 m/s	4	0.154	−0.233	0.540	33.2	0.436	5.48	3	0.140	45.2
	< 0.8 m/s	9	0.829	0.312	1.346	66.8	0.002	37.77	8	< 0.001	78.8
Resistance training modalities											
Work load intensity**	< 70%1RM	7	0.843	0.222	1.464	40.7	0.008	25.74	6	< 0.001	76.7
	≥70% 1RM	8	0.580	0.185	0.975	51.8	0.004	20.72	7	0.004	66.2
	ND	1	0.000	−0.566	0.566	7.5	–	–	0	–	–
Frequency, sessions/week	3	11	0.669	0.272	1.067	78.0	0.001	42.69	10	< 0.001	76.6
	2	2	0.515	−0.566	1.596	14.3	0.350	4.79	1	0.029	79.1
	7	1	0.000	−0.566	0.566	7.8	–	–	0	–	–
Study quality parameters											
Allocation concealment	Yes	6	0.352	0.011	0.693	43.7	0.043	9.84	5	0.08	49.2
	No	8	0.761	0.207	1.316	56.3	0.007	39.00	7	< 0.001	82.1
Groups were similar at baseline	Yes	11	0.506	0.102	0.911	78.1	0.014	45.64	10	< 0.001	78.1
	No	3	0.873	0.515	1.231	21.9	< 0.001	0.88	2	0.645	0.0
Subjects were blinded	Yes	7	0.651	0.107	1.195	51.9	0.019	37.25	6	< 0.001	83.9
	No	7	0.522	0.109	0.935	48.1	0.013	14.86	6	0.021	59.6
Assessors were blinded	Yes	11	0.421	0.150	0.639	78.8	0.002	20.84	10	0.022	52.0
	No	3	1.128	−0.215	2.472	21.2	0.100	23.90	2	< 0.001	96.6
End point data on 85% of the participants was obtained	Yes	7	0.743	0.194	1.293	51.2	0.008	35.24	6	< 0.001	83.0
	No	7	0.409	0.028	0.791	48.8	0.036	13.63	6	0.034	56.0
Intention-to-treat analysis were performed	Yes	8	0.764	0.191	1.337	56.8	0.009	42.75	7	< 0.001	83.6
	No	6	0.349	0.065	0.633	43.3	0.016	6.86	5	0.231	27.2

*Results by the derSimonian and Laird random-effects method using Hedges’ g

**In this sub-analysis two studies [17, 45] are represented by two intervention groups that exercised at different intensities

*SMD* Standardised Mean Difference, *CI* Confidence Intervals, *d.f.* degrees of freedom, *Q* Heterogeneity statistics, *I*^*2*^ the variation in SMD attributable to heterogeneity, *CD* Community-Dwelling, *GI* Geriatric Institution, *SH* sheltered housing, *ND* no data, *RM* repetition maximum, *m/s* meter per second

In addition to the subgroup analysis, meta-regressions were performed to explore the differences in treatment effect by participant characteristics (mean age as a continuous variable, and gait speed at baseline as a categorical variable < 0.8 or ≥ 0.8 m/second) and by the RT-modalities (load intensity (%1RM, continuous) and duration (weeks, continuous)). Two studies [17, 45] had two intervention groups that only differed by load intensity. These groups were separately represented in the meta-regression regarding RT-load intensity. Intervention duration and mean age were significantly associated with the effects on self-reported disability in that shorter duration and higher age predicted greater effects (duration: coefficient − 0.074, *p* = 0.024, Adj. R^2^ = 65.1%, I^2^ res. = 44.9%, *n* = 13 studies; and age: coefficient 0.088, *p* = 0.027 Adj. R^2^ = 43.7%, I^2^ res. = 64.8%, *n* = 14 studies, Table 6).

**Table 6:** Results from meta-regressions

Covariate	Coeffi-cient	Standard error	95% lower CI	95% upper CI	t-value	*p*-value	Adj.R^2^%	I^2^ res., %	N
Participant characteristics									
Mean age (years, continuous)	0.088	0.03	0.01	0.16	2.51	0.027	43.7	64.8	14
Gait speed at baseline (< 0.8 / ≥0.8 m*s^−1^)	0.679	0.43	−0.27	1.63	1.58	0.143	13.4	75.6	13
Resistance training program modality									
Program duration (weeks, continuous)	−0.074	0.03	−0.14	−0.01	−2.62	0.024	65.1	44.9	13
^a^Work load intensity (% 1RM, continuous)	−0.019	0.01	−0.05	0.01	−1.61	0.134	18.2	68.6	14
Resistance training effects on									
Knee-extensor strength (SMD)	0.341	0.24	−0.22	0.91	1.43	0.196	18.1	78.1	9
Lower body functional capacity (SMD)	0.772	0.13	0.49	1.06	6.06	< 0.001	99.2	0.0	12
Gait capacity (SMD)	0.365	0.23	−0.17	0.90	1.61	0.152	57.5	32.9	9

^a^For the load intensity covariate two studies [17, 45] are represented by two intervention groups that exercised at different intensities

*SMD* standardised mean difference from random effects model using Hedges’ g

#### Association between effects on objective study outcomes and self-reported ADL–disability

**Fig. 4:**
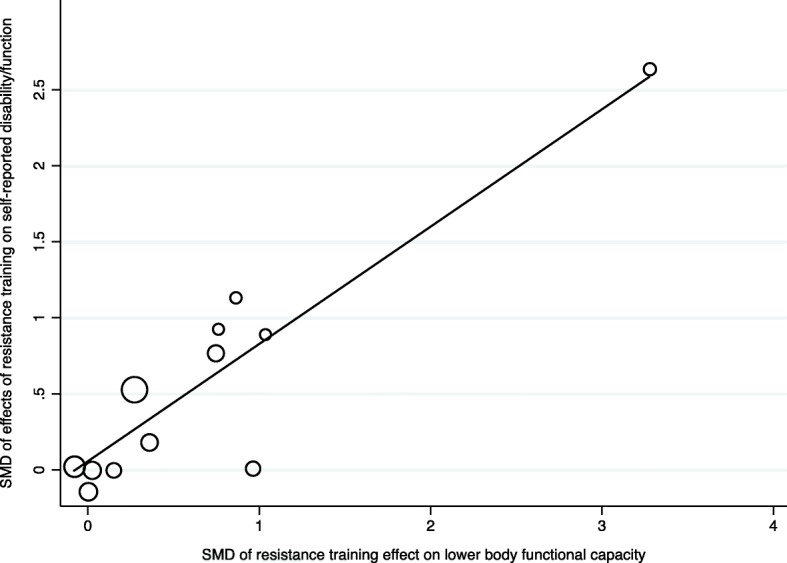
Association between self-reported disability and lower body functional capacity. Bubble-plot from meta-regression on the association between self-reported disability and lower body functional capacity. Dependent factor: Standardised mean differences (SMD) of resistance training effects on self-reported disability. Covariate: SMDs of the effect of resistance training on lower body functional capacity

The random effects meta-analyses on KE-strength (*n* = 9) and lower body functional capacity (*n* = 12) revealed significant positive effects of RT of large (SMD = 0.97) and moderate (SMD = 0.63) size respectively (Table 4). The effect on gait capacity was small (SMD = 0.36) and did not reach significance (9 studies). Heterogeneity was moderate or high in all three analyses. The effects of RT on KE-strength and gait capacity were not associated with change in self-reported ADL-disability (*p* = 0.196, and 0.152 respectively). However, effects on lower body functional capacity was significantly associated with SMD in self-reported disability (coefficient: 0.772, 95% CI: 0.49 to 1.06; *p* < 0.001, Adj. R^2^ = 99.2%, I^2^_res_ = 0.0%, *n* = 12 studies, Fig. 4). An overview of results from meta-regressions are presented in Table 6.

## Discussion

### Summary of evidence

This systematic review and meta-analysis showed that resistance training has a significant moderate positive effect on self-reported disability in older adults with pre-existing functional limitations or disability. This evidence was based in 14 trials investigating the effect of resistance training on self-reported function or disability related to ADL and IADL.

The quality of the studies was generally high, with 11 out of 14 trials scoring ≥6 on the PEDro scale. There was a trend for publication bias, but this was caused by a single medium-size study. Meta-regressions performed on a subset of the studies, revealed that there was: i) a strong association between improvements in lower body functional capacity objectively assessed (e.g. chair-rise) and reduced self-reported disability (12 trials), ii) no association between reduced self-reported disability and either improvements in knee extensor strength or gait capacity (9 trials each). Higher age predicted greater effects on self-reported disability. In general, RT seems to be a safe method in this population. Transient inconveniencies (i.e. muscle soreness and exacerbation of musculoskeletal conditions such as arthritis) are the most frequently reported cases, and serious adverse events appear to be rare (two injuries reported in 1422 participants).

### Primary results

The eligibility criteria that were applied in this study increase participant homogeneity in terms of age (≥65 years) and functional state at baseline compared to earlier meta-analysis [23]. This may explain the four-fold larger effect on self-reported disability seen in the current meta-analysis compared with the earlier Cochrane review by Liu and Latham (SMD 0.59 vs. SMD 0.14) [23]. Poor performance in functional tests objectively assessed (e.g. gait, chair-rise or balance) is a well-known predictor of future disability in ADL [61–63]. Therefore, in populations with no or only subtle functional limitation, the baseline levels of perceived ADL-function will expectedly be high, and improvements following a strength training intervention may be difficult to detect [6]. Keysor & Jette [22] addressed the inadequacies of the existing tools to measure perceived disability already more than 15 years ago in a systematic review of exercise effects on disability. These authors stressed that low responsiveness and ceiling and floor effects were major shortcomings in the self-reported ADL-disability outcomes (amongst others the Barthel Index and the SF-36). Despite the two most frequent outcomes in the present review were the Barthel Index and the SF-36, ceiling effects were only discussed as an issue in one of the articles included [35]. Only six studies stated that the selected tool was validated, and the validations were not necessarily performed in the same population (i.e. older adults with functional limitation or disability) or for the same purpose (i.e. detecting the effects of a RT intervention).

The supplementary vote-count analysis allowed us to include six additional studies. This additional analysis indicated that there is no effect of strength training on self-reported measures of disability. This is in opposition to the moderate positive effects shown by the meta-analysis without these six additional studies. However, the result of the vote-count analysis must be interpreted with caution, as this procedure does not take into account the quality of the studies, the sample size, or the size and variation of the effects in the individual studies. Also, one major advantage of a meta-analysis over the vote-count procedure, is indeed the increased power to show significant effects by pooling of data from several smaller studies.

Nevertheless, this indicates that studies with negative results may more frequently report selective or incomplete data and the primary results of the present study could potentially be biased by this in a positive direction.

#### Associations between changes in self-reported disability and objectively measured function following RT

##### Lower body functional capacity

The meta-analysis on lower body functional capacity showed significant moderate effect (12 studies, SMD 0.625, *p* = 0.002) of a similar magnitude as the meta-analysis on self-reported disability. Moreover, the meta-regression between these two outcomes revealed that the size of the effect of RT programs on lower body functional capacity almost completely explained the heterogeneity in effects on self-reported disability across studies (Adj. R^2^ = 99.2%). Visual inspection of the bubble-plot of this meta-regression (Fig. 4), indicates that one study may be the main cause of this nearly perfect linear relationship. Therefor a robustness analysis, omitting that study [45] from the meta-regression, was performed. In the new analysis without this study, the association was still significant (*p* = 0.025), the proportion of between study heterogeneity explained was high (85.84%) and the residual variation due to heterogeneity was minimal (3.78%). The strong association between RT effects on self-reported function and lower body functional capacity is a novel finding. It is surprising because it is in contrast with previous meta-analyses finding large effects of RT programs on objective measures of function but small effect on self-reported disability.

##### Knee extensor strength

The effect size for KE-strength was large (9 studies, SMD 0.97, *p* < 0.001). This is in agreement with previous studies in healthy older adults [12] and in older adults not specifically characterized by functional limitation or disability [23], indicating that changes in muscle strength may be independent of pre-existing limitations or disability, and that for muscle strength ceiling effect may not be an issue. The present study found no association between RT effects on KE-strength and self-reported disability. Muscle power of the lower limbs has been shown to be stronger associated with functional capacity than muscle strength [64], and recent studies suggest that muscle power and strength of the hip muscles in particular is linked to balance control in older adults [65]. Because muscle power may be a stronger predictor of future disability, studies investigating the changes in muscle power and self-report disability following RT programs may provide valuable information. Nevertheless, data available for a meta-regression on this specific association were sparse and it was not the aim of this meta-analysis.

##### Gait capacity

In the current study, the effect of RT on gait was not statistically significant (9 studies, SMD = 0.357, *p* = 0.061). This finding is in contrast with previous meta-analyses which demonstrated large significant effects of RT on gait capacity in older adults who were not selected by functional limitation- or disability criteria [23, 66]. Gait capacity was a secondary outcome in the present review, and the articles eligible for the meta-analysis on gait capacity were restricted to be only those including both self-reported disability and an objective measure of gait. The purpose of performing this secondary meta-analysis was to use the calculated SMDs in a subsequent meta-regression. This was also the case for the meta-analyses on KE-strength and lower body functional capacity. Because of that methodological aspect the meta-analyses on gait capacity, KE-strength and lower body functional capacity do not represent comprehensive systematic literature searches on these three outcomes and should not be interpreted as such. This may also account for the difference in RT effects on gait in the present study from previous literature.

Meta-regression revealed no association between gait speed and self-reported disability. This may be explained by the fact that the self-reported tools cover a broader set of aspects of function and disability, which are better reflected in lower body functional capacity than in gait capacity. One could argue, that it would have been more suitable to investigate associations between self-reported mobility and gait capacity. However, since gait speed is a well-known predictor of incident disability [61], we found it relevant to specifically investigate if increases in gait speed are associated with improvements in self-reported disability.

##### Investigations of heterogeneity

This study specifically aimed at investigating the effect of resistance training in older adults with pre-existing functional limitations or disability.

This possibly increases the external validity and the generalizability of our results for this target group, compared to previous meta-analyses which included both well-functioning and functionally limited older adults. The effect of resistance training on older adults with pre-existing limitations or disability are less likely to be affected by ceiling effect for self-report assessment of disability and may translate in more clinically meaningful benefits than for their higher-functioning peers.

Nevertheless, the moderate positive effect on self-reported disability observed in this study was associated with statistical heterogeneity (Table 4). This was not significantly reduced when the data from trials utilizing different training modalities or features of the study design (e.g. blinding) were pooled separately in sub-group meta-analyses. The number of studies in each of the subgroups was low. This impairs the strength of these sub-group analyses and the conclusions that can be drawn from them. When meta-regressions to investigate the influence of duration and load intensity of training as well as participant characteristics (age and gait speed at baseline) were performed we found that shorter duration and higher age were associated with larger effects. Contrary to what has been found in meta-analysis investigating the effects of RT on muscle strength [12, 23, 67] there was no evidence in this data that load intensity predicts the effects of RT on self-reported disability. Possibly, changing the perception of disability in older adults is not fully mediated by muscle function (e.g. muscle strength or power), indicating a more complex interplay between various components, that is still to be uncovered and understood by further research.

### Strengths and limitations

To our knowledge, this is the first systematic review to investigate associations between objectively measured performance-based outcomes and self-reported disability on a study-level using meta-regressions in older adults with pre-existing functional limitation or disability.

A limitation of these meta-regressions is that they were performed on a low number of studies (ranging from 9 to 12 studies). The probability of finding a positive association by chance alone, is higher when running multiple sub-analysis in a meta-analysis study with a low number of studies. However, the results of the various additional analysis do highlight factors that may be important in understanding the heterogeneity of effects and designate questions that need to be addressed in further research.

A novel finding was the good methodological quality of the included studies. Most of the studies (19 out of 20), however, failed to satisfy at least one of the applied quality criterions that are known to increase internal validity (i.e. intention-to-treat analysis, blinded outcome assessors, attention control groups (subject blinding), or allocation concealment). Sub-analysis showed no evidence, that studies not fulfilling these criteria systematically over- or underestimated the effects. One study [40] had a score of 3 on the PEDro scale. The low-quality rating of this trial did not affect the results of the meta-analysis and meta-regressions, as this trial has been excluded from these analyses for other reasons.

The most frequently unsatisfied quality criterion was *“missing follow-up data from more than 15% of the initially allocated participants”*, which might be due to selective drop-out because of very old age (i.e. mean age was > 80 years in 60% of the included studies). For this population, critical illness and death is to be expected even during shorter intervention periods. A clear example of this is the result from a study where more than 15% of the initially allocated participants were lost to death alone [36]. In spite of that, the mean drop-out rate across studies in this systematic review was lower than what has been reported in exercise studies in general [68]. Another strength of the present systematic review is that eligible studies, which could not be included in the meta-analysis because of analysis design or incomplete reporting of data, were still addressed in the review. This feature adds valuable information to the statistical results increasing the transparency of the evidence provided. The characteristics of the articles that were eligible for vote-count analysis only did not differ from those included in the statistical meta-analysis. Also, the mean PEDro quality score and the proportion of studies below the threshold of six on the PEDro scale was similar between included and non-included studies.

## Conclusion

Based on the current evidence, RT has moderate positive effects on self-reported disability in older adults with functional limitations and or self-reported disability. Shorter duration of the RT-intervention as well as higher age predicted greater effects. Additionally, gains in lower body functional capacities are associated with positive effects on self-reported disability. However, no such association was found regarding gait capacity or muscle strength.

The results demonstrate that the continuously growing population of older adults at risk for or with existing ADL-disability, can benefit from RT in terms of improvements in self-reported ADL-function. Moreover, the diversity in intervention modalities and settings evident from these data, support that implementation of effective RT interventions is feasible and can be incorporated into routine health care services.

The finding of a clear association between effects in objective lower body functional capacity and self-reported function/disability supports the relevance of objective tests of physical function to evaluate RT intervention effectiveness also in terms of detecting changes that are perceivable for the individual. In this review the associations of effects on different outcomes were investigated on a study-level. The material and data synthesized here, provides no insight into how and by which factors such association may be moderated or modified in individuals. We call for more research to investigate these relationships with approaches allowing for investigation of direction, potential modifiers and moderators of interactions. Gaining more knowledge about such underlying mechanisms is imperative to enable optimization of future exercise interventions in producing effects that are clinically meaningful to the individual and society.

## Supplementary information

**Additional file 1:** Structure of the search strategy, and the exact terms searched in each database. Detailed search strategy and list of terms and subject headings searched in each of the bibliographic databases PubMed, Embase, Web of Science, SPORTDiscus and CINAHL.

**Additional file 2:** Overview of primary outcome measures. Self-reported disability (Table 1A). Overview of secondary outcome measures. Objective measures of muscle function and functional capacity (Table 2A). Effects of resistance training on measures of self-reported disability/function including outlier. Forest plot from meta-analysis of the effect of resistance training interventions on self-reported disability or function in older adults including the study by Fahlman et al. (Fig. 1a).

## Data Availability

Not applicable.

## Abbreviations

ADL: Activities of Daily Living
BI: Barthel Index
CG: Control Group
CI: Confidence Interval
Corr: Correlation estimate
d.f.: degrees of freedom
IADL: Instrumental Activities of Daily Living
ICF: International Classification of Functioning, Disability and Health
IG: Intervention Group
KE-strength: Knee Extensor strength
PEDro: Physiotherapy Evidence Database
PFD: Physical Function Domain
PICOS: Participants, Intervention, Comparison, Outcomes, Study design
RCT: Randomized Controlled Trial
RM: Repetition Maximum
RPE: Rate of Perceived Exertion
RT: Resistance Training
SD: Standard Deviation
SE: Standard Error
SF-36: 36-Item Short Form Health Survey Instrument
SMD: Standardized Mean Difference
WHO: World Health Organisation

## References

[1] Wahrendorf M, Reinhardt JD, Siegrist J (2013). Relationships of disability with age among adults aged 50 to 85: evidence from the United States, England and continental europe. PLoS One.

[2] United Nations, D.o.E.a.S.A., Population Division (2015). World population Ageing 2015 - Highlights.

[3] European Commission, The 2015 Ageing Report. Economic and budgetary projections for the 28 EU Member States (2013–2060), Economic Policy Committee, Editor. 2015: European Economy.

[4] Organization, W.H (2001). International classification of functioning, disability and health: ICF.

[5] Samuel D (2012). The relationships between muscle strength, biomechanical functional moments and health-related quality of life in non-elite older adults. Age Ageing.

[6] Guralnik JM, Patel K, Ferrucci L, Newman AB, Cauley JA (2012). Assessing Functional Status and Disability in Epidemiologic Studies, in The epidemiology of aging.

[7] Fried TR (2001). Functional disability and health care expenditures for older persons. Arch Intern Med.

[8] World Health Organization (2012). Strategy and action plan for healthy ageing in Europe, 2012–2020.

[9] Chodzko-Zajko WJ (2009). American College of Sports Medicine position stand. Exercise and physical activity for older adults. Med Sci Sports Exerc.

[10] Aagaard P (2010). Role of the nervous system in sarcopenia and muscle atrophy with aging: strength training as a countermeasure. Scand J Med Sci Sports.

[11] Caserotti P (2008). Explosive heavy-resistance training in old and very old adults: changes in rapid muscle force, strength and power. Scand J Med Sci Sports.

[12] Borde R, Hortobagyi T, Granacher U (2015). Dose-response relationships of resistance training in healthy old adults: a systematic review and meta-analysis. Sports Med.

[13] Valenzuela T (2012). Efficacy of progressive resistance training interventions in older adults in nursing homes: a systematic review. J Am Med Dir Assoc.

[14] Latham N, et al. Progressive resistance strength training for physical disability in older people. Cochrane Database Syst Rev. 2003;(2):Cd002759. 10.1002/14651858.CD002759 12804434

[15] Binder EF (2002). Effects of exercise training on frailty in community-dwelling older adults: results of a randomized, controlled trial. J Am Geriatr Soc.

[16] Chandler JM (1998). Is lower extremity strength gain associated with improvement in physical performance and disability in frail, community-dwelling elders?. Arch Phys Med Rehabil.

[17] Seynnes O (2004). Physiological and functional responses to low-moderate versus high-intensity progressive resistance training in frail elders. J Gerontol A Biol Sci Med Sci.

[18] Westhoff MH, Stemmerik L, Boshuizen HC (2000). Effects of a low-intensity strength-training program on knee-extensor strength and functional ability of frail older people. J Aging Phys Act.

[19] Binder EF (2004). Effects of extended outpatient rehabilitation after hip fracture: a randomized controlled trial. JAMA.

[20] Services, U.S.D.o.H.a.H. Physical Activity Guidelines for Americans. 2nd ed: U.S. Washington, DC: Department of Health and Human Services; 2018.

[21] Brochu M (2002). Effects of resistance training on physical function in older disabled women with coronary heart disease. J Appl Physiol (1985).

[22] Keysor JJ, Jette AM (2001). Have we oversold the benefit of late-life exercise?. J Gerontol A Biol Sci Med Sci.

[23] Liu CJ, Latham NK. Progressive resistance strength training for improving physical function in older adults. Cochrane Database Syst Rev. 2009;3(3). 10.1002/14651858.CD002759.pub2 PMC4324332 19588334

[24] Weening-Dijksterhuis E (2011). Frail institutionalized older persons: a comprehensive review on physical exercise, physical fitness, activities of daily living, and quality-of-life. Am J Phys Med Rehabil.

[25] Liberati A (2009). The PRISMA statement for reporting systematic reviews and meta-analyses of studies that evaluate health care interventions: explanation and elaboration. J Clin Epidemiol.

[26] Moher D (2009). Preferred reporting items for systematic reviews and meta-analyses: the PRISMA statement. J Clin Epidemiol.

[27] Medicine, C.f.E.-B (2016). Asking Focused Questions.

[28] Elkins MR (2010). Rating the quality of trials in systematic reviews of physical therapy interventions. Cardiopulm Phys Ther J.

[29] Mangione KK (2005). Can elderly patients who have had a hip fracture perform moderate- to high-intensity exercise at home?. Phys Ther.

[30] Jette AM (2009). Toward a common language of disablement. J Gerontol A Biol Sci Med Sci.

[31] Veritas Health Innovation, M., Australia (2016). Covidence systematic review software.

[32] Ades PA (2003). Resistance training on physical performance in disabled older female cardiac patients. Med Sci Sports Exerc.

[33] Benavent-Caballer V (2014). Effects of three different low-intensity exercise interventions on physical performance, muscle CSA and activities of daily living: a randomized controlled trial. Exp Gerontol.

[34] Boshuizen HC (2005). The effects of physical therapists' guidance on improvement in a strength-training program for the frail elderly. J Aging Phys Act.

[35] Buchner DM (1997). The effect of strength and endurance training on gait, balance, fall risk, and health services use in community-living older adults. J Gerontol A Biol Sci Med Sci.

[36] Cadore EL (2014). Multicomponent exercises including muscle power training enhance muscle mass, power output, and functional outcomes in institutionalized frail nonagenarians. Age.

[37] Chin APMJM (2006). Once a week not enough, twice a week not feasible?. A randomised controlled exercise trial in long-term care facilities [ISRCTN87177281]. Patient Educ Couns.

[38] Clemson L (2012). Integration of balance and strength training into daily life activity to reduce rate of falls in older people (the LiFE study): Randomised parallel trial. BMJ (Online).

[39] Danilovich M (2016). The impact of strong for life on the physical functioning and health of older adults receiving home and community-based services. Aging Soc.

[40] Fahlman M (2007). Combination training and resistance training as effective interventions to improve functioning in elders. J Aging Phys Act.

[41] Hewitt J (2018). Progressive resistance and balance training for falls prevention in long-term residential aged care: a cluster randomized trial of the sunbeam program. J Am Med Dir Assoc.

[42] Latham NK (2003). A randomized, controlled trial of quadriceps resistance exercise and vitamin D in frail older people: the frailty interventions trial in elderly subjects (FITNESS). J Am Geriatr Soc.

[43] McMurdo MET, Johnstone R (1995). A randomized controlled trial of a home exercise programme for elderly people with poor mobility. Age Ageing.

[44] Mihalko SL, McAuley E (1996). Strength training effects on subjective well-being and physical function in the elderly. J Aging Phys Act.

[45] Sahin Ulku K., Kirdi Nuray, Bozoglu Ergun, Meric Aydin, Buyukturan Galip, Ozturk Ahmet, Doruk Huseyin (2018). Effect of low-intensity versus high-intensity resistance training on the functioning of the institutionalized frail elderly. International Journal of Rehabilitation Research.

[46] Timonen L (2006). Effects of group-based exercise program on functional abilities in frail older women after hospital discharge. Aging Clin Exp Res.

[47] Venturelli M (2010). Positive effects of physical training in activity of daily living-dependent older adults. Exp Aging Res.

[48] Brzycki M (1993). Strength testing—predicting a one-rep max from reps-to-fatigue. J Phys Educ Recreation Dance.

[49] Maher CG (2003). Reliability of the PEDro scale for rating quality of randomized controlled trials. Phys Ther.

[50] Fu R, Vandermeer BW, Shamliyan TA, O’Neil ME, Yazdi F, Fox SH, Morton SC (2013). Handling Continuous Outcomes in Quantitative Synthesis. Methods Guide for Comparative Effectiveness Reviews.

[51] Higgins JPT, G S (2011). Cochrane Handbook for Systematic Reviews of Interventions Version 5.1.0 [updated March 2011].

[52] Higgins, J.P. and S.G. Thompson, Quantifying heterogeneity in a meta-analysis*.* (0277–6715 (Print)). 10.1002/sim.1186 12111919

[53] Harris R (2008). Metan: fixed- and random-effects meta-analysis. Stata J.

[54] Higgins JP (2003). Measuring inconsistency in meta-analyses. Bmj.

[55] Harbord RM, Higgins JPT (2008). Meta-regression in Stata. Stata J.

[56] Peters JL (2008). Contour-enhanced meta-analysis funnel plots help distinguish publication bias from other causes of asymmetry. J Clin Epidemiol.

[57] Egger M, Smith DG, Schneider M, Minder C (1997). Bias in meta-analysis ditected by a simple, graphical test. Br Med J.

[58] Bushman BJ, Wang MC, Cooper LVHH, Valentine JC (2009). Vote-counting procedures in meta-analysis, in The handbook of research synthesis and meta-analysis.

[59] Drey M (2012). Effects of strength training versus power training on physical performance in prefrail community-dwelling older adults. Gerontology.

[60] Mahoney FI, Barthel DW (1965). Functional evaluation: the BARTHEL index. Md State Med J.

[61] Abellan van Kan G (2009). Gait speed at usual pace as a predictor of adverse outcomes in community-dwelling older people an International Academy on Nutrition and Aging (IANA) Task Force. J Nutr Health Aging.

[62] Guralnik JM (2000). Lower extremity function and subsequent disability: consistency across studies, predictive models, and value of gait speed alone compared with the short physical performance battery. J Gerontol A Biol Sci Med Sci.

[63] Perera S (2016). Gait speed predicts incident disability: a pooled analysis. J Gerontol A Biol Sci Med Sci.

[64] Reid KF, Fielding RA (2012). Skeletal muscle power: a critical determinant of physical functioning in older adults. Exerc Sport Sci Rev.

[65] Porto JM (2019). Contribution of hip abductor-adductor muscles on static and dynamic balance of community-dwelling older adults. Aging Clin Exp Res.

[66] Hortobagyi T (2015). Effects of three types of exercise interventions on healthy old Adults' gait speed: a systematic review and meta-analysis. Sports Med.

[67] Csapo R, Alegre LM (2016). Effects of resistance training with moderate vs heavy loads on muscle mass and strength in the elderly: a meta-analysis. Scand J Med Sci Sports.

[68] Linke SE, Gallo LC, Norman GJ (2011). Attrition and adherence rates of sustained vs. intermittent exercise interventions. Ann Behav Med.

[69] Liu-Ambrose TY (2005). Both resistance and agility training reduce back pain and improve health-related quality of life in older women with low bone mass. Osteoporos Int.

[70] Maher CG (2000). A systematic review of workplace interventions to prevent low back pain. Aust J Physiother.

