# Supplementary material for: Effects of resistance training on self-reported disability in older adults with functional limitations or disability – a systematic review and meta-analysis
Source: Eur Rev Aging Phys Act. 2019 Dec 7;16:24. doi: 10.1186/s11556-019-0230-5 (PMC6898935; doi:10.1186/s11556-019-0230-5)
Supplement: Supplementary file 1 — Additional file 1: Structure of the search strategy, and the exact terms searched in each database. Detailed search strategy and list of terms and subject headings searched in each of the bibliographic databases PubMed, Embase, Web of Science, SPORTDiscus and CINAHL. [file 11556_2019_230_MOESM1_ESM.docx]

# **Additional file 1**

### Structure of the search strategy, and the exact terms searched in each database

Table 1 Search strategy for the PubMed database

| Set 1 (term 1-75) are the **Text Words** (TW) [words found in the title, abstract, MeSH headings and Subheadings, Other Terms field of a record] for the **population** (i.e. older adults)  The term “aged” is searched exclusively as a MeSH term (i.e. not as a Text Word) in order to avoid excessive noise.  All terms are combined using **OR** | Set 1   1. aging 2. elderly 3. senior 4. seniors 5. frail elderly 6. nonagenarian 7. nonagenarians 8. old adult 9. old adults 10. old individual 11. old individuals 12. old men 13. old women 14. old people 15. old person 16. old persons 17. old patient 18. old patients 19. old subject 20. old subjects 21. old female 22. old females 23. old male 24. old males 25. older adult 26. older adults 27. older individual 28. older individuals 29. older men 30. older women 31. older people 32. older person 33. older persons 34. older patient 35. older patients 36. older subject 37. older subjects 38. older female 39. older females 40. older male 41. older males 42. geriatric adult 43. geriatric adults 44. geriatric individual 45. geriatric individuals 46. geriatric men 47. geriatric women 48. geriatric people 49. geriatric person 50. geriatric persons 51. geriatric patient 52. geriatric patients 53. geriatric subject 54. geriatric subjects 55. geriatric female 56. geriatric females 57. geriatric male 58. geriatric males 59. Aged adult 60. Aged adults 61. Aged individual 62. Aged individuals 63. Aged men 64. Aged women 65. Aged people 66. Aged person 67. Aged persons 68. Aged patient 69. Aged patients 70. Aged subject 71. Aged subjects 72. Aged female 73. Aged females 74. Aged male 75. Aged males 76. Aged MeSH | **Population (P)** |
| --- | --- | --- |
| Set 2 (term 77-90) are the **Text Words** (TW) [words found in the title, abstract, MeSH headings and Subheadings, Other Terms field of a record] for the **Intervention** (i.e. resistance training). They are combined using **OR** | Set 2   1. weight lifting 2. progressive strength training 3. progressive resistance training 4. weight exercise 5. weight training 6. power exercise 7. power training 8. strengthening exercise 9. strength exercise 10. strength training 11. resistive exercise 12. resistance exercise 13. resistive training 14. resistance training | **Intervention (P)** |
| Set 3 (term 91-112), 4 (term 113-115) and 5 (term 116-124) are the **Text Words** (TW) [words found in the title, abstract, MeSH headings and Subheadings, Other Terms field of a record] for the **outcome** (i.e. self-reported disability/function).  Set 4 and 5 is combined by AND in set 8.  Set 3 and 6 are combined using **OR** | Set 3   1. activities of daily living 2. ADL 3. IADL 4. BADL 5. PADL 6. health related quality of life 7. HRQoL 8. quality of life 9. QOL 10. questionnair* 11. self-rated 12. self-report* 13. disab* 14. physical health status 15. functional fitness 16. functional gain 17. late life disability index 18. late life function index 19. oars 20. gars 21. health assessment quesionnaire 22. nhanes adl   set 4   1. physical* 2. funct* 3. Mobility   Set 5   1. independen* 2. dependen* 3. limit* 4. abilit* 5. funct* 6. autonomy 7. perform* 8. capacit* 9. impair*   set 6   1. set 4 **AND** set 5 | **Outcome (O)** |
| Set 7 (term 126-128), are the terms searched in **Title field** (TI) [words found in the TITLE of a record] for the **Study designs** that are **excluded from this review** (i.e. reviews, meta-analysis and case-reports). They are combined using **OR** | Set 7   1. review 2. meta analysis 3. case report | **Study Design (SD)** |
| Set 8 is the combination of  **Population (P), intervention (I) & outcome (O)**. They are combined by **AND**  Set 9 is the combination of set 8 and 7 combined by **NOT** [the combined search of P, I and O restricted from studies mentioning a study-designs, which is excluded in the review, in its title) | Set 8   1. P **AND** I **AND** O   Set 9   1. Set 8 **NOT** set 7 | **Combination of  P & I & O & SD** |

Table 2 Search strategy for the Embase database

| Set 1 (term 1-75) are the terms searched in **the multiple purpose field (mp)** [words found in the title, abstract, heading word, drug trade name, original title, device manufacturer, drug manufacturer, device trade name, keyword, floating subheading word, candidate term word of a record] for the **population** (i.e. older adults)  Set 2 (term 76-81) are the **Subject Terms** for the **population** (i.e. older adults).    They are combined used **OR** | Set 1   1. aging 2. elderly 3. senior 4. seniors 5. frail elderly 6. nonagenarian 7. nonagenarians 8. old adult 9. old adults 10. old individual 11. old individuals 12. old men 13. old women 14. old people 15. old person 16. old persons 17. old patient 18. old patients 19. old subject 20. old subjects 21. old female 22. old females 23. old male 24. old males 25. older adult 26. older adults 27. older individual 28. older individuals 29. older men 30. older women 31. older people 32. older person 33. older persons 34. older patient 35. older patients 36. older subject 37. older subjects 38. older female 39. older females 40. older male 41. older males 42. geriatric adult 43. geriatric adults 44. geriatric individual 45. geriatric individuals 46. geriatric men 47. geriatric women 48. geriatric people 49. geriatric person 50. geriatric persons 51. geriatric patient 52. geriatric patients 53. geriatric subject 54. geriatric subjects 55. geriatric female 56. geriatric females 57. geriatric male 58. geriatric males 59. Aged adult 60. Aged adults 61. Aged individual 62. Aged individuals 63. Aged men 64. Aged women 65. Aged people 66. Aged person 67. Aged persons 68. Aged patient 69. Aged patients 70. Aged subject 71. Aged subjects 72. Aged female 73. Aged females 74. Aged male 75. Aged males   Set 2   1. aged/ 2. aging/ 3. very elderly/ 4. frail elderly/ 5. geritrics/ 6. geriatric patients/ | **Population (P)** |
| --- | --- | --- |
| Set 3 (term 82-95) are the terms searched in **the multiple purpose field (mp)** [words found in the title, abstract, heading word, drug trade name, original title, device manufacturer, drug manufacturer, device trade name, keyword, floating subheading word, candidate term word of a record] for the i**ntervention** (i.e. resistance training).  Set 4 (term 96) is the **Subject Term** for the **Intervention** (i.e. resistance training).  All terms are combined using **OR** | Set 3   1. weight lifting 2. progressive strength training 3. progressive resistance training 4. weight exercise 5. weight training 6. power exercise 7. power training 8. strengthening exercise 9. strength exercise 10. strength training 11. resistive exercise 12. resistance exercise 13. resistive training 14. resistance training   Set 4   1. resistance training/ | **Intervention (P)** |
| Set 5 (term 97-118), 6 (term 119-121) and 7 (term 122-130) are the terms searched in **the multiple purpose field (mp)** [words found in the title, abstract, heading word, drug trade name, original title, device manufacturer, drug manufacturer, device trade name, keyword, floating subheading word, candidate term word of a record] for the **outcome** (i.e. self-reported disability/function).  Set 6 and 7 is combined by AND in set 8.  Set 9 (term 131-134) are the **Subject Terms** for the **outcome** (i.e. self-reported disability/function.  Set 5, 8 and 9 are combined using **OR** | Set 5   1. activities of daily living 2. ADL 3. IADL 4. BADL 5. PADL 6. health related quality of life 7. HRQoL 8. quality of life 9. QOL 10. questionnair* 11. self-rated 12. self-report* 13. disab* 14. physical health status 15. functional fitness 16. functional gain 17. late life disability index 18. late life function index 19. oars 20. gars 21. health assessment quesionnaire 22. nhanes adl   set 6   1. physical* 2. funct* 3. Mobility   Set 7   1. independen* 2. dependen* 3. limit* 4. abilit* 5. funct* 6. autonomy 7. perform* 8. capacit* 9. impair*   set 8  set 6 AND set 7  set 9   1. “quality of life"/ 2. “quality of life assessment"/ 3. “quality of life index"/ 4. exp “daily life activity”/ | **Outcome (O)** |
| Set 10 is the combination of  **Population (P), intervention (I) & outcome (O)**. They are combined using **AND** | Set 11  P **AND** I **AND** O | **Combination of  P & I & O** |

Table 3 Search strategy for the Web of Science database

| Set 1 (term 1-75) are the terms searched in **the Topic field** [words found in the title, abstract, author keywords, and Keywords Plus of a record] for the **population** (i.e. older adults)  They are combined used **OR** | Set 1   1. aging 2. elderly 3. senior 4. seniors 5. frail elderly 6. nonagenarian 7. nonagenarians 8. old adult 9. old adults 10. old individual 11. old individuals 12. old men 13. old women 14. old people 15. old person 16. old persons 17. old patient 18. old patients 19. old subject 20. old subjects 21. old female 22. old females 23. old male 24. old males 25. older adult 26. older adults 27. older individual 28. older individuals 29. older men 30. older women 31. older people 32. older person 33. older persons 34. older patient 35. older patients 36. older subject 37. older subjects 38. older female 39. older females 40. older male 41. older males 42. geriatric adult 43. geriatric adults 44. geriatric individual 45. geriatric individuals 46. geriatric men 47. geriatric women 48. geriatric people 49. geriatric person 50. geriatric persons 51. geriatric patient 52. geriatric patients 53. geriatric subject 54. geriatric subjects 55. geriatric female 56. geriatric females 57. geriatric male 58. geriatric males 59. Aged adult 60. Aged adults 61. Aged individual 62. Aged individuals 63. Aged men 64. Aged women 65. Aged people 66. Aged person 67. Aged persons 68. Aged patient 69. Aged patients 70. Aged subject 71. Aged subjects 72. Aged female 73. Aged females 74. Aged male 75. Aged males | **Population (P)** |
| --- | --- | --- |
| Set 2 (term 77-90) are the terms searched in **the Topic field** [words found in the title, abstract, author keywords, and Keywords Plus of a record] for the i**ntervention** (i.e. resistance training).  They are combined using **OR** | Set 2   1. weight lifting 2. progressive strength training 3. progressive resistance training 4. weight exercise 5. weight training 6. power exercise 7. power training 8. strengthening exercise 9. strength exercise 10. strength training 11. resistive exercise 12. resistance exercise 13. resistive training 14. resistance training | **Intervention (P)** |
| Set 3 (term 90-111), 4 (term 112-114) and 5 (term 115-123) are the terms searched in **the Topic field** [words found in the title, abstract, author keywords, and Keywords Plus of a record] for the **outcome** (i.e. self-reported disability/function). Set 4 and 5 is combined by AND in set 6.  Set 3 and 6 are combined using **OR** | Set 3   1. activities of daily living 2. ADL 3. IADL 4. BADL 5. PADL 6. health related quality of life 7. HRQoL 8. quality of life 9. QOL 10. questionnair* 11. self-rated 12. self-report* 13. disab* 14. physical health status 15. functional fitness 16. functional gain 17. late life disability index 18. late life function index 19. oars 20. gars 21. health assessment quesionnaire 22. nhanes adl   set 4   1. physical* 2. funct* 3. Mobility   Set 5   1. independen* 2. dependen* 3. limit* 4. abilit* 5. funct* 6. autonomy 7. perform* 8. capacit* 9. impair*   set 6   1. set 4 AND set 5 | **Outcome (O)** |
| Set 7 (term 125-127), are the terms searched in **the Topic field** [words found in the title, abstract, author keywords, and Keywords Plus of a record] for the **Study designs** that are **excluded from this review** (i.e. reviews, meta-analysis and case-reports). They are combined using **OR** | Set 7   1. review 2. meta analysis 3. case report | **Study Design (SD)** |
| Set 8 is the combination of  **Population (P), intervention (I) & outcome (O)**. They are combined by **AND**  Set 9 is the combination of set 8 and 7 combined by **NOT** [the combined search of P, I and O restricted from studies mentioning a study-designs, which is excluded in the review, in its title) | Set 8   1. P **AND** I **AND** O   Set 9   1. Set 8 **NOT** set 7 | **Combination of  P & I & O & SD** |

Table 4 Search strategy for the SPORTDiscus and CINAHL databases

| Set 1 (term 1-75) are the terms searched in **Title** (TI) and **Abstract** Fields (AB) [words found in the TITLE or ABSTRACT of a record] for the **population** (i.e. older adults)  Set 2 (term 76-79) are the **Subject Terms (SU)** for the **population** (i.e. older adults). They are combined used **OR** | Set 1   1. aging 2. elderly 3. senior 4. seniors 5. frail elderly 6. nonagenarian 7. nonagenarians 8. old adult 9. old adults 10. old individual 11. old individuals 12. old men 13. old women 14. old people 15. old person 16. old persons 17. old patient 18. old patients 19. old subject 20. old subjects 21. old female 22. old females 23. old male 24. old males 25. older adult 26. older adults 27. older individual 28. older individuals 29. older men 30. older women 31. older people 32. older person 33. older persons 34. older patient 35. older patients 36. older subject 37. older subjects 38. older female 39. older females 40. older male 41. older males 42. geriatric adult 43. geriatric adults 44. geriatric individual 45. geriatric individuals 46. geriatric men 47. geriatric women 48. geriatric people 49. geriatric person 50. geriatric persons 51. geriatric patient 52. geriatric patients 53. geriatric subject 54. geriatric subjects 55. geriatric female 56. geriatric females 57. geriatric male 58. geriatric males 59. Aged adult 60. Aged adults 61. Aged individual 62. Aged individuals 63. Aged men 64. Aged women 65. Aged people 66. Aged person 67. Aged persons 68. Aged patient 69. Aged patients 70. Aged subject 71. Aged subjects 72. Aged female 73. Aged females 74. Aged male 75. Aged males   Set 2   1. Aged [SU] 2. elderly [SU] 3. aged, 80 and over [SU] 4. frail elderly [SU] | **Population (P)** |
| --- | --- | --- |
| Set 3 (term 80-93) are the terms searched in **Title** (TI) and **Abstract** Fields (AB) [words found in the TITLE or ABSTRACT of a record] for the **Intervention** (i.e. resistance training).  Set 4 (term 94-95) are the **Subject Terms (SU)** for the **Intervention** (i.e. resistance training). They are combined using **OR** | Set 3   1. weight lifting 2. progressive strength training 3. progressive resistance training 4. weight exercise 5. weight training 6. power exercise 7. power training 8. strengthening exercise 9. strength exercise 10. strength training 11. resistive exercise 12. resistance exercise 13. resistive training 14. resistance training   Set 4   1. SU exercise therapy 2. SU resistance training | **Intervention (P)** |
| Set 5 (term 96-117), 6 (term 118-120) and 7 (term 121-129) are the terms searched in **Title** (TI) and **Abstract** Fields (AB) [words found in the TITLE or ABSTRACT of a record] for the **outcome** (i.e. self-reported disability/function). Set 6 and 7 is combined by AND in set 8.  Set 9 (term 130-134) are the **Subject Terms** (SU) for the **outcome** (i.e. self-reported disability/function.  Set 5, 8 and 9 are combined using **OR** | Set 5   1. activities of daily living 2. ADL 3. IADL 4. BADL 5. PADL 6. health related quality of life 7. HRQoL 8. quality of life 9. QOL 10. questionnair* 11. self-rated 12. self-report* 13. disab* 14. physical health status 15. functional fitness 16. functional gain 17. late life disability index 18. late life function index 19. oars 20. gars 21. health assessment quesionnaire 22. nhanes adl   set 6   1. physical* 2. funct* 3. Mobility   Set 7   1. independen* 2. dependen* 3. limit* 4. abilit* 5. funct* 6. autonomy 7. perform* 8. capacit* 9. impair*   set 8  set 6 AND set 7  set 9   1. SU activities of daily living 2. SU quality of life 3. SU geriatric functional assessment 4. SU functional assessment 5. SU questionnaires | **Outcome (O)** |
| Set 10 (term 135-137), are the terms searched in **Title** (TI) [words found in the TITLE of a record] for the **Study designs** that are **excluded from this review** (i.e. reviews, meta-analysis and case-reports). They are combined using **OR** | Set 10   1. review 2. meta analysis 3. case report | **Study Design (SD)** |
| Set 11 is the combination of  **Population (P), intervention (I) & outcome (O)**. They are combined by **AND**  Set 12 is the combination of set 10 and 11 combined by **NOT** [the combined search of P, I and O restricted from studies mentioning a study-designs, which is excluded in the review, in its title) | Set 11  P **AND** I **AND** O  Set 12  Set 11 **NOT** set 10 | **Combination of  P & I & O & SD** |
