# Supplementary material for: Effects of resistance training on self-reported disability in older adults with functional limitations or disability – a systematic review and meta-analysis
Source: Eur Rev Aging Phys Act. 2019 Dec 7;16:24. doi: 10.1186/s11556-019-0230-5 (PMC6898935; doi:10.1186/s11556-019-0230-5)
Supplement: Supplementary file 2 — Additional file 2: Overview of primary outcome measures. Self-reported disability (Table 1A). Overview of secondary outcome measures. Objective measures of muscle function and functional capacity (Table 2A). Effects of resistance training on measures of self-reported disability/function including outlier. Forest plot from meta-analysis of the effect of resistance training interventions on self-reported disability or function in older adults including the study by Fahlman et al. (Fig. 1a). [file 11556_2019_230_MOESM2_ESM.docx]

# **Additional file 2**

Additional file 2, table 1A Primary outcome measures. Self-reported disability

| **Instruments used to assess self-reported ADL-function/disability** | **Study** | **Facet/**  **dimension** | **Name of subscale (when relevant)** | **Scale** |
| --- | --- | --- | --- | --- |
| BI  *the Barthel Index* | Benavent-Caballer et al., 2014  Cadore et al., 2014  Venturelli et al., 2010  Sahin et al., 2018 | Need for assistance |  | 0-100  Higher is better |
|  | McMurdo & Johnstone, 1995 |  |  | 0-20  Higher is better |
| MOS SF-36  *The Medical Outcome Study 36-item Short Form Survey* | Ades et al., 2003  Chandler et al., 1998*  Hewitt et al., 2018*  Latham et al., 2003* | Physical limitation | Physical function | 0-100  Higher is better |
|  | Fahlman et al., 2007* |  |  | Scale not reported^$^  Higher is better |
| The Lawton and Brody Instrumental Activities of Daily Living Scale | Mihalko & McAuley, 1996 | Likert scale  Cannot 🡪can easily do | 20 selected items out of 25 | 20-140  Higher is better |
|  | Buchner et al., 1997 | Number of independent IADLs | Selected items: Transportation, cooking, shopping, housework, laundry | 0-5  Higher is better |
| GARS  *the Groningen Activity Restriction Scale (ADL/IADL)* | Boshuizen et al., 2005 | Difficulty,  Need for assistance |  | 18-72  Lower is better |
|  | Westhoff, Stemmerick & Boshuizen, 2000 |  | Lower extremity specific | 6-18  Lower is better |
| FSQ  *Functional Status Questionnaire* | Binder et al., 2002 | Difficulty | ADL | 0-36  Higher is better |
| Disability in 17 ADLs | Chin a Pow et al., 2006 | Difficulty/ Need for assistance |  | 0-51  Higher is better |
| The National Health and Nutrition Examination  Surveys (NHANES) activity of daily living  NHANES ADL-instrument  *The National Health and Nutrition Examination Surveys independence measure for Activities of Daily Living* | Clemson et al., 2012* | Difficulty |  | Scale not reported |
| PROMIS  *Patient-Reported Outcome Measurement Information System* | Danilovich et al., 2016 | Miscellaneous  Functional ability | Physical summary  ADL | (Scale not reported)  1-5  Higher is better |
| HAQ-DI  *Health Assessment Questionnaire* | Seynnes et al., 2004 | Difficulty | Disability Index | 0-3^§^  Lower is better |
| Joensuu classification of ADL/IADL skills* | Timonen et al., 2006* | Miscellaneous |  | 3 categories |

^$^ Based on the values, we assume that this scale does not go from 0 to 100. ^§^ The final score is an average of the scores (0-3) in 8 categories of ADL. * Scale and/or trial is not included in primary meta-analysis of summed SMDs. ADL=activities of daily living, IADL= instrumental activities of daily living

Additional file 2, table 2A Secondary outcome measures. Objective measures of muscle function and functional capacity

| **Instruments applied for measuring knee-extensor strength** | **Studies included in meta-regression** | **Method** | **Type of contraction** |
| --- | --- | --- | --- |
| Knee extensor strength | Cadore et al., 2014  Seynnes et el., 2004 | One repetition maximum | Dynamic |
|  | Boshuizen et al., 2005  Chin a Pow et al., 2006  Danilovich et al., 2016  Westhoff, Stemmerick & Boshuizen 2000  Sahin et al., 2018 | Maximal Voluntary Contraction | Isometric |
|  | Binder et al., 2002  Buchner et al., 1997 | Maximal Voluntary Contraction | Isokinetic |
| **Instruments applied for measuring lower body functional capacity** | **Studies included in meta-regression** | **Components** | **Domain** |
| CS-PFP  Continuous-Scale Physical Function Performance test | Ades et al., 2003 | Not specified | Lower body |
| PPT  Physical Performance Test  Modified version | Binder et al., 2002 | Walking; coat on/off; pick up penny from floor;  chair-rise; lifting item to shelf; stair climb, balance |  |
| TUG  Timed Up-and-Go | Benavent-Caballer et al., 2014  Boshuizen et al., 2005  Cadore et al., 2014  McMurdo & Johnstone 1995  Danilovich et al., 2016  Westhoff, Stemmerick & Boshuizen 2000 | Chair-rise, walk, turn |  |
| SPPB  Short Physical Performance Battery | Sahin et al., 2018 | Chair-rise, balance, walk |  |
| Chair-rise | Chin a Pow et al., 2006 | Chair-rise Five repetitions |  |
|  | Seynnes et el., 2004 | Chair-rise n repetitions in 30 seconds |  |
| Stair climb | Buchner et al., 1997 | 11 steps at comfortable pace |  |
| **Instruments applied for measuring gait capacity** | **Studies included in meta-regression** | **Outcomes** | **Distance** |
| 6MWT  Six Minutes Walk Test | Ades et al., 2003  Benavent-Caballer et al., 2014  Seynnes et al., 2004 | Distance covered in a set time |  |
| Set distance walk test | Boshuizen et al., 2005  Westhoff, Stemmerick & Boshuizen 2000 | Time spend to cover a set distance (speed) | 20 meters  (2 x 10 and a turn) |
|  | Cadore et al., 2014 |  | 5 meters (+ ac/de-celeration) |
|  | Buchner et al., 1997 |  | 40 meters |
|  | Chin a Pow et al., 2006 |  | 8 meters |
|  | Danilovich et al., 2016 |  | 10 feet |

Forrest plot from meta-analysis of the effect of resistance training interventions on self-reported disability or function in older adults including the study by Fahlman et al[1]. Results are from random effects model using Hedges’ g. SMD=standardised mean difference; N=number of participants

Additional file 2 figure 1A Effects of resistance training on measures of self-reported disability/function. Including outlier

1. Fahlman, M., et al., *Combination training and resistance training as effective interventions to improve functioning in elders.* Journal of Aging and Physical Activity, 2007. **15**(2): p. 195-205.
